# Supplementary figures and images for: Determinants of immunisation dropout among children under the age of 2 in Zambézia province, Mozambique: a community-based participatory research study using Photovoice
Source: BMJ Open. 2022 Mar 15;12(3):e057245. doi: 10.1136/bmjopen-2021-057245 (PMC8928306; doi:10.1136/bmjopen-2021-057245)

**Appendix A: Adapted Increasing Vaccination Model**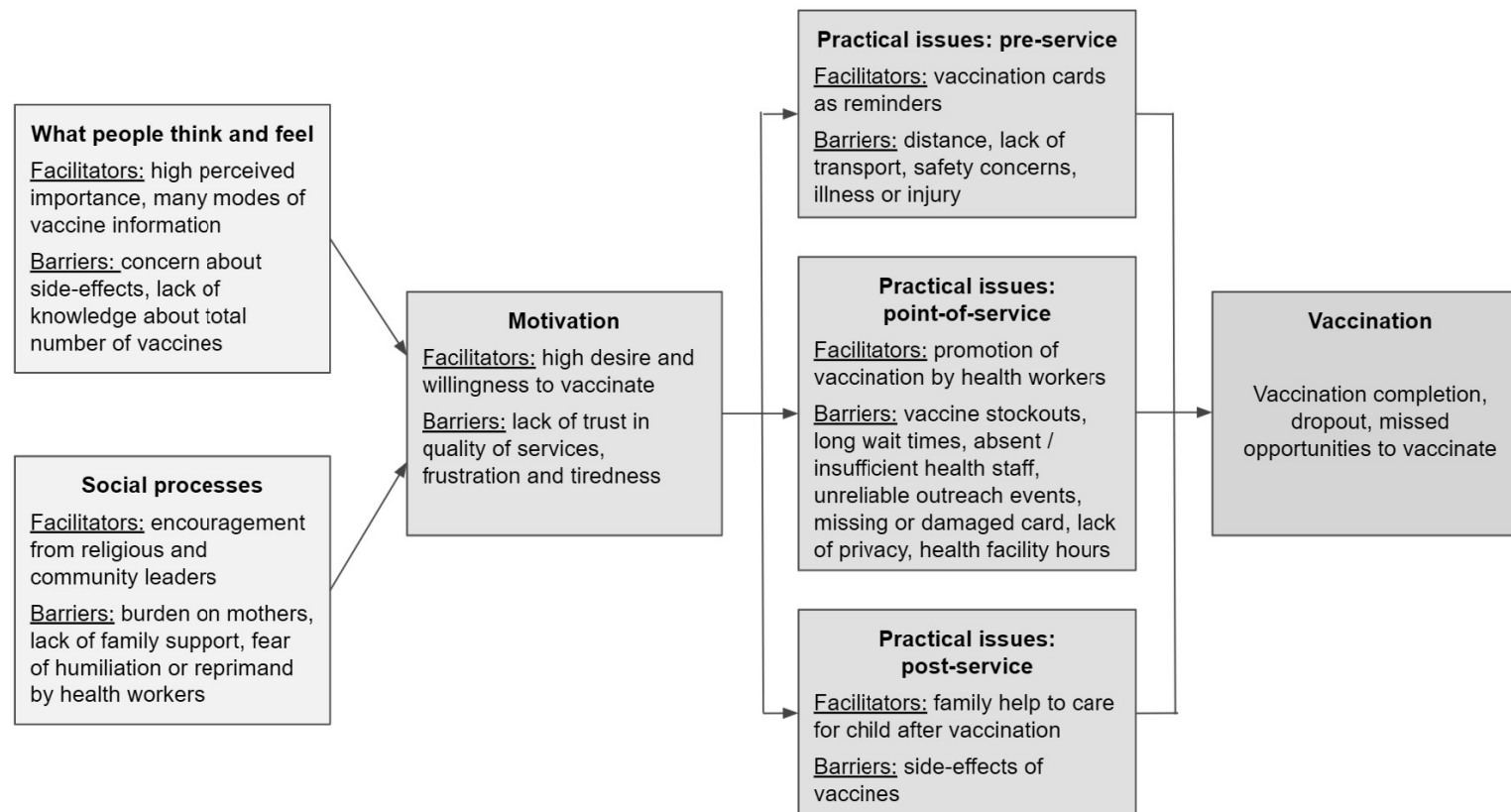

Supplement: Supplementary data [file bmjopen-2021-057245supp001.pdf]
