# Supplementary material for: Determinants of immunisation dropout among children under the age of 2 in Zambézia province, Mozambique: a community-based participatory research study using Photovoice
Source: BMJ Open. 2022 Mar 15;12(3):e057245. doi: 10.1136/bmjopen-2021-057245 (PMC8928306; doi:10.1136/bmjopen-2021-057245)
Supplement: Supplementary data [file bmjopen-2021-057245supp002.pdf]

Appendix B: Mozambique Childhood Immunization Schedule

**Vaccine**	**Dose**	**Age of administration per Mozambique EPI schedule**	
		**Ideal**	**Allowed Range**
Tuberculosis	BCG	Birth	Birth – 23 months
Oral Polio	OPV 0	Birth	Birth – 6 weeks
	OPV 1	2 months	6 weeks – 23 months
	OPV 2	3 months	10 weeks – 23 months
	OPV 3	4 months	14 weeks – 23 months
Pentavalent: Diphtheria, Pertussis, Tetanus, Hepatitis B, Haemophilus influenzae type B	DPT-HepB-Hib 1	2 months	6 weeks – 23 months
	DPT-HepB-Hib 2	3 months	10 weeks – 23 months
	DPT-HepB-Hib 3	4 months	14 weeks – 23 months
Pneumococcal conjugate	PCV 1	2 months	6 weeks – 23 months
	PCV 2	4 months	14 weeks – 23 months
	PCV 3	9 months	9 months – 23 months
Rotavirus	RV 1	2 months	6 weeks – 23 months
	RV 2	3 months	10 weeks – 14 weeks
Inactivated Polio	IPV	4 months	14 weeks – 23 months
Measles, Rubella	MR 1	9 months	9 months – 23 months
	MR 2	18 months	18 months – 23 months
