# Supplementary material for: Determinants of immunisation dropout among children under the age of 2 in Zambézia province, Mozambique: a community-based participatory research study using Photovoice
Source: BMJ Open. 2022 Mar 15;12(3):e057245. doi: 10.1136/bmjopen-2021-057245 (PMC8928306; doi:10.1136/bmjopen-2021-057245)
Supplement: Supplementary data [file bmjopen-2021-057245supp003.pdf]

Appendix C: Semi-Structured Interview Guide for Caregiver Interviews

Introduction: Good morning/afternoon. I am back to go over the photographs and conduct the remainder of the interview with you. Is now still a good time?

Introduction to in-depth interview:

- This interview will last about 1.5 hours.
- Please know that what you say to us is confidential. We know how important this is to you. We also ask the other participants keep our discussions confidential.
- We would like to record the conversation so that we can document all the information you provide.
- To better understand immunization in your community, it is really important that you share your true beliefs and attitudes towards vaccination, as well as your personal experiences in vaccination of your children.

Informed consent for photos and recording the interview: Now before we get started, I would like to request your informed consent again to review the photographs and record the interview. As mentioned last week, your participation is voluntary and there is no penalty for not taking part. You may refuse to answer any question or withdraw from the study at any time.
Do you have any questions or would you like me to read the informed consent again?

If agree, have them sign the lines on the consent forms for the photographs and recording of the interview!

Discuss "Ground Rules" for the In-depth Interview

- Please don't answer your cell-phone during this interview.
- Do you have any other questions before we get started?

Review of Photovoice:

- Ask them to show the photos they have taken on the camera
- Ask them to pick 3-5 photos that best represent their experience of immunizing [child's name].
- Make sure to pick photographs that are clear!
- **Note: If there are any photos of them, their child or family members, request a signed photo release form by each individual in the photo! If it is the child, they can sign for their child.**
- Upload the selected photos to the computer and paste them in PowerPoint and begin the photovoice session and interview.
    - If the computer isn't working, review the photos on the digital camera and note down the photo number for future reference
- Turn on the recorder!
- Begin the interview using the interview guide

Semi-Structured Discussion Guide for Photovoice In-depth Interview

Instructions: To elicit rich descriptions from participants, when asking follow-up questions or encouraging them to talk more or give you more examples/details, use the following probes:

- *What do you mean by ____ ?*
- *Tell me more about ____.*
- *Can you give me an example of ____ ?*
- *Can you tell me about a time when ____ ?*
- *Can you tell me about the last time ____ ?*
- *Is there anything that made ____ easier?*
- *Is there anything that made ____ harder?*
- *(Can you tell me) Who ____?*
- *(Can you tell me) When ____ ?*
- *(Can you tell me) Where ____ ?*

Probes are completed using only verbatim participant words or phrases.

Interview Guide:
Before we discuss the photos more, can you tell us about how the photovoice process went for you? Did you discuss the task with anyone else in your family or community or have any help in taking the photos?
If yes, Did they have any influence on any of the photos you took?

- If so, in what way?
- Can you provide an example of how they impacted the photos you took and selected?

Once photos are selected, ***ask for each photo:***

- What do we see in this photo?
- What does this represent to you?
- How does this photo represent your vaccination journey?

Now, based on our activity with the photos, we have learned more about your experience, thank you. Now we have some more questions to better understand your experience immunizing your child and the things that influenced [child's name] immunization status. Do you have your child's immunization card which we can refer to during this interview?

1) To start, can you tell me about how people in your community talk about immunizations?
   a) How does this relate to how you feel about immunizing [child's name]?
   b) Has this changed at all with COVID-19?
      i) If yes, why and how?
      ii) If no, why not?

2) Can you describe to me what the experience was like getting immunizations for [child's name]?
   a) How many times did [child's name] get immunizations before the age of two? (look at immunization card)?

b) When did they get these immunizations?
    i) [if they got immunizations more than once] Were these experiences the same? Different?
    ii) How so?
c) Where did [child's name] get their immunizations?
    i) Did they get them all there?
    ii) What did you think about getting the immunizations there?
d) Who immunized [child's name]?
    i) How do you feel about that?
    ii) Did you trust the [health worker/nurse/doctor] who gave the immunizations?
        (1) Why or why not?
e) How satisfied were you with the experience(s) of immunizing [child's name]?
    i) What lead to you feeling this satisfied/dissatisfied?

3) When deciding to immunize [child's name], what things did you consider?
    a) Was there anyone who helped you make that decision?
        i) Who? How did they help?
    b) Did you intend for your child to be fully vaccinated according to the recommended schedule?
        i) If yes, why?
        ii) If no, why not?
    c) Did you have questions about the recommendations for immunizing [child's name]?
        i) Why or why not?
    d) How important did you feel getting these immunizations was, if at all, for [child's name]?
    e) Have things changed since COVID-19?
        i) If so, how?

4) What kind of information, if at all, did you receive about immunizing your child or about immunizations in general?
    a) When did you receive this information?
    b) Where did this information come from?
        i) Health center?
        ii) Social media (Facebook, WhatsApp)?
        iii) Neighbours?
    c) How often, if at all, did you receive information?
    d) To what extent did you trust this information?
    e) Did you get information about how many immunizations your child needed and when they were needed?
    f) Did this change with COVID-19? How?

5) [for those who have a child who dropped out] Can you describe why [child's name] didn't receive all their vaccinations by age two?
   a) What factors do you think contributed to that?
      i) Probe on:
         (1) What kind of information did you receive about your child's immunization schedule, if at all?
         (2) Did you know what your child's immunization schedule was?
         (3) COVID-19
   b) What was hard about trying to immunize [child's name]?
      i) Can you provide an example?
      ii) How, if at all, has this changed since COVID-19?
   c) What was easy about trying to immunize [child's name]?
      i) Can you provide an example?
      ii) How, if at all, has this changed since COVID-19?

6) [for those who have a fully-immunized child] What factors do you think contributed to being able to fully immunize [child's name] by the age of two?
   a) What was hard about trying to immunize [child's name]?
      i) Can you provide an example?
      ii) How, if at all, has this changed since COVID-19?
   b) What was easy about trying to immunize [child's name]?
      i) Can you provide an example?
      ii) How, if at all, has this changed since COVID-19?

7) How do you wish the experience of getting all the recommended immunizations for [child's name] could be easier, if at all?
   a) What would you change?
   b) What would you keep the same?
   c) How would these changes look in times where there is no COVID-19?
   d) How does it look now with COVID-19?

Conclusion: Are there any final thoughts you wish to share about your experiences with immunizing your 2-3 year old child?

We would like to thank you for taking the time to discuss this important topic with us!
