# Supplementary material for: Determinants of immunisation dropout among children under the age of 2 in Zambézia province, Mozambique: a community-based participatory research study using Photovoice
Source: BMJ Open. 2022 Mar 15;12(3):e057245. doi: 10.1136/bmjopen-2021-057245 (PMC8928306; doi:10.1136/bmjopen-2021-057245)
Supplement: Supplementary data [file bmjopen-2021-057245supp004.pdf]

Appendix D: Semi-Structured Interview Guide for Health Worker Interviews

Introduction to Structured Phone Interview: The purpose of the debrief activities is to reflect on and discuss your messages with the healthcare workers you corresponded with. You will use the message notes to debrief with the healthcare workers about the immunization process. This is a rough guide of questions to ask the healthcare workers, and additional questions or topics can be discussed based on specific observations that you made from the messages.

Interview Questions

1) Now, I am going to walk through some of the activities you mentioned in your messages, and I would like to get your perspective on:
    a) Were these typical [experiences, processes, observations]?
        i) If so, how? If not, how did they vary from normal?
    b) Is there anything else you would like to share or explain about the [experience, process, observation] that may not have been captured in the messages?

2) How do you see your role in immunizations?
    a) How do you feel about that role?
    b) Do you feel supported in your role? If not, why? If yes, how so?
    c) How do you generally feel about the tasks associated with immunizations?

3) How do you feel about how immunization records are reported?
    a) How do you feel about overall system for immunizations?
    b) What about management of immunizations?

4) Now, I would like to get a better sense of your work around immunizing children under the age of two. Can you walk through a typical day of conducting immunizations for children under the age of two? [Probe on what activities they do to prepare for immunizations, conduct immunizations, document immunizations, educate around immunization]
    For each step/activity?
    a) Why do you take that particular step?
    b) Who is typically involved in that step?
    c) What you feel when going through that step?
    d) What factors make that step easier/harder?
    e) Can you guide me through the steps that you took today when administering a vaccination?
    f) Are there any cases where you would not administer a vaccination to a child? Why or why not?

5) Are there other, non-typical ways that you provide immunizations?
    a) Can you tell me about those?

6) Outside of conducting immunizations, what else do you do?
    a) Can you tell me about how under-two vaccinations fit into this workload?

7) What is challenging about administering under-two vaccinations?
    a) Did you encounter any of those challenges over the past month? If so, which ones? How did that make your job more challenging?

8) What factors make it easier for you to administer vaccinations?
    a) Did you encounter any of those factors over the past month? If so, which ones? How did that make your job easier?

9) What are your personal beliefs around vaccinations?

10) Can you describe the conversations you usually have with caregivers while administering vaccines?
    a) What about how you see your role in providing advice or convincing caregivers to vaccinate their children under the age of two?

11) What do you view as the primary drivers of under-two immunization drop-outs?
    a) Can you provide an example?
