# Supplementary material for: Determinants of immunisation dropout among children under the age of 2 in Zambézia province, Mozambique: a community-based participatory research study using Photovoice
Source: BMJ Open. 2022 Mar 15;12(3):e057245. doi: 10.1136/bmjopen-2021-057245 (PMC8928306; doi:10.1136/bmjopen-2021-057245)

Appendix E: Photovoice Instructional Materials

A CÂMERA

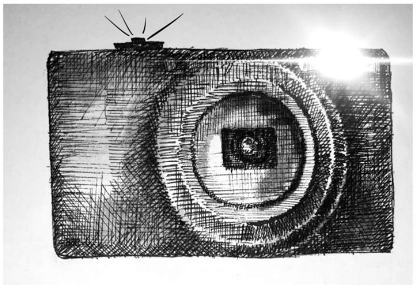

1. Frente da câmera

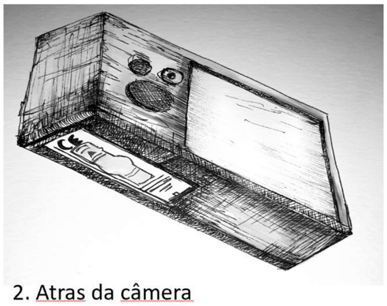

2. Atras da câmera

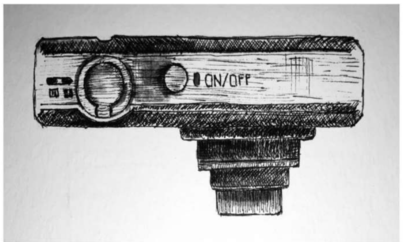

3. Parte superior da câmera

SEGURAR A CÂMERA PARA TIRAR PHOTOS

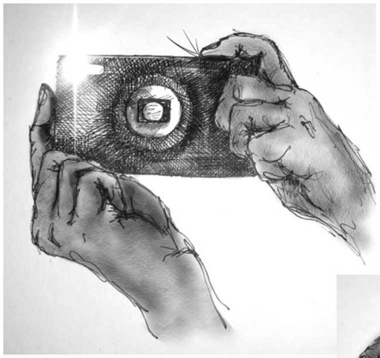

1. Uma Maneira

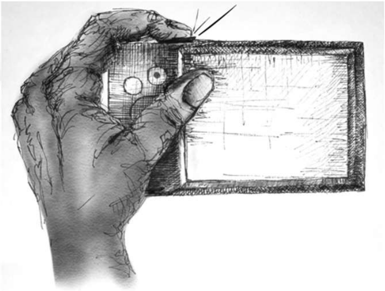

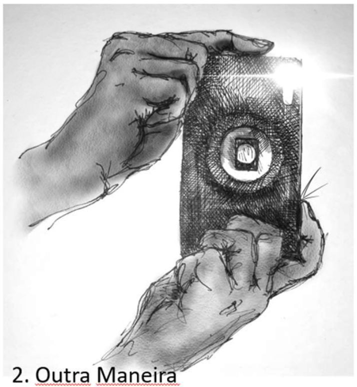

2. Outra Maneira

BATERIA DA CÂMERA

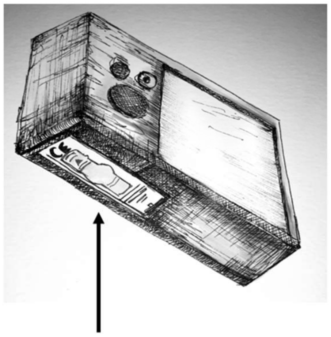

1. Localização da bateria

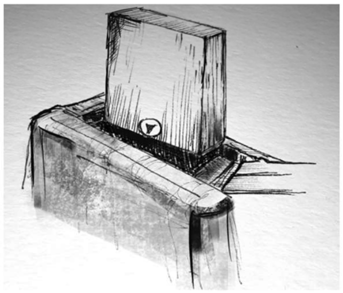

2. Abra o local da bateria

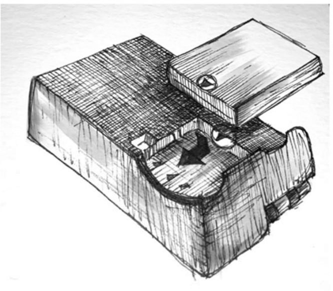

3. Carrega a bateria no carregador

TIRE FOTOS DO SEU AMBIENTE QUE REPRESENTAM A SUA EXPERIENCIA DE VACCINACAO DA SU CRIANCA

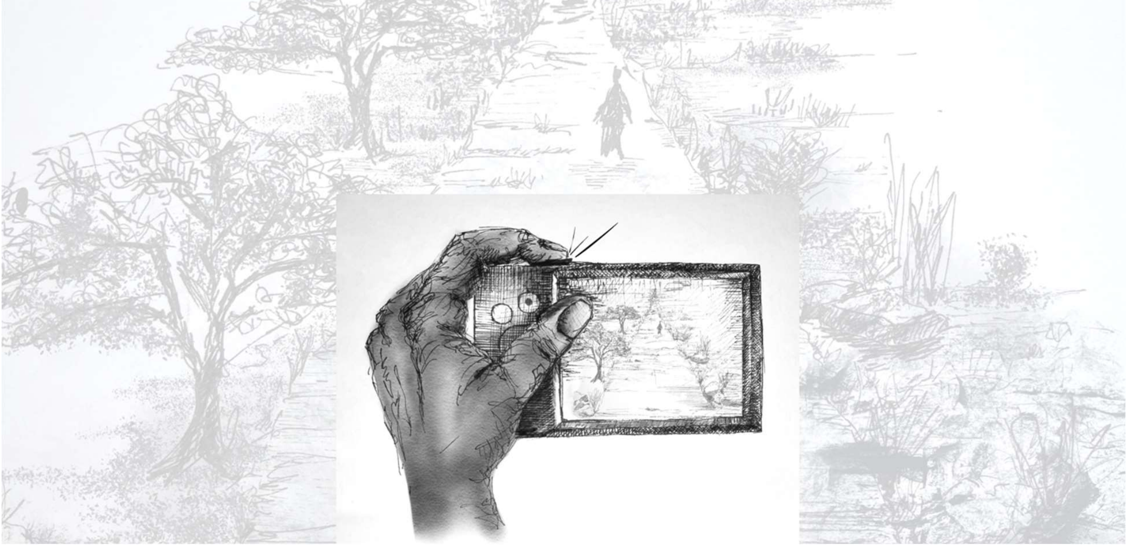

Supplement: Supplementary data [file bmjopen-2021-057245supp005.pdf]
