# Supplementary material for: Determinants of immunisation dropout among children under the age of 2 in Zambézia province, Mozambique: a community-based participatory research study using Photovoice
Source: BMJ Open. 2022 Mar 15;12(3):e057245. doi: 10.1136/bmjopen-2021-057245 (PMC8928306; doi:10.1136/bmjopen-2021-057245)
Supplement: Author's manuscript [file bmjopen-2021-057245.draft_revisions.pdf]

BMJ Open is committed to open peer review. As part of this commitment we make the peer review history of every article we publish publicly available.

When an article is published we post the peer reviewers' comments and the authors' responses online. We also post the versions of the paper that were used during peer review. These are the versions that the peer review comments apply to.

The versions of the paper that follow are the versions that were submitted during the peer review process. They are not the versions of record or the final published versions. They should not be cited or distributed as the published version of this manuscript.

BMJ Open is an open access journal and the full, final, typeset and author-corrected version of record of the manuscript is available on our site with no access controls, subscription charges or pay-per-view fees (http://bmjopen.bmj.com).

If you have any questions on BMJ Open's open peer review process please email info.bmjopen@bmj.com

BMJ Open

Determinants of immunization dropout among children under the age of two in Zambézia Province, Mozambique: A community-based participatory research study using Photovoice

Journal:	*BMJ Open*
Manuscript ID	bmjopen-2021-057245
Article Type:	Original research
Date Submitted by the Author:	28-Oct-2021
Complete List of Authors:	Powelson, Jocelyn; VillageReach<br>Magadzire, Bvudzai; VillageReach<br>Draiva, Abel; VillageReach<br>Denno, Donna; University of Washington, Department of Pediatrics<br>Ibraimo, Abdul; VillageReach<br>Benate, Bonifácia; Independent<br>Jahar, Lídia; Independent<br>Marrune, Zélia; Independent<br>Chilundo, Baltazar; VillageReach<br>Chinai, Jalilo; Zambézia Provincial Directorate of Health<br>Emerson, Michelle; VillageReach<br>Beima-Sofie, Kristin; University of Washington School of Public Health, Department of Global Health<br>Lawrence, Emily; VillageReach
Keywords:	PUBLIC HEALTH, QUALITATIVE RESEARCH, International health services < HEALTH SERVICES ADMINISTRATION & MANAGEMENT

SCHOLARONE™
Manuscripts

I, the Submitting Author has the right to grant and does grant on behalf of all authors of the Work (as defined in the below author licence), an exclusive licence and/or a non-exclusive licence for contributions from authors who are: i) UK Crown employees; ii) where BMJ has agreed a CC-BY licence shall apply, and/or iii) in accordance with the terms applicable for US Federal Government officers or employees acting as part of their official duties; on a worldwide, perpetual, irrevocable, royalty-free basis to BMJ Publishing Group Ltd ("BMJ") its licensees and where the relevant Journal is co-owned by BMJ to the co-owners of the Journal, to publish the Work in this journal and any other BMJ products and to exploit all rights, as set out in our licence.

The Submitting Author accepts and understands that any supply made under these terms is made by BMJ to the Submitting Author unless you are acting as an employee on behalf of your employer or a postgraduate student of an affiliated institution which is paying any applicable article publishing charge ("APC") for Open Access articles. Where the Submitting Author wishes to make the Work available on an Open Access basis (and intends to pay the relevant APC), the terms of reuse of such Open Access shall be governed by a Creative Commons licence – details of these licences and which Creative Commons licence will apply to this Work are set out in our licence referred to above.

Other than as permitted in any relevant BMJ Author's Self Archiving Policies, I confirm this Work has not been accepted for publication elsewhere, is not being considered for publication elsewhere and does not duplicate material already published. I confirm all authors consent to publication of this Work and authorise the granting of this licence.

For peer review only

Determinants of immunization dropout among children under the age of two in Zambézia Province, Mozambique: A community-based participatory research study using Photovoice

Authors

Jocelyn Powelson, VillageReach, Seattle, USA

Bvudzai Magadzire, VillageReach, Cape Town, South Africa

Abel Draiva, VillageReach, Quelimane, Mozambique

Donna M. Denno, Department of Pediatrics, University of Washington, Seattle, USA

Abdul Ibraimo, VillageReach, Maputo, Mozambique

Bonifácia Beleza Lucas Benate, Independent, Namarroi, Mozambique

Lídia Carlos Jahar, Independent, Gilé, Mozambique

Zélia Marrune, Independent, Gilé, Mozambique

Baltazar Chilundo, VillageReach, Maputo, Mozambique

Jalilo Ernesto Chinai, Zambézia Provincial Directorate of Health, Quelimane, Mozambique

Michelle Emerson, VillageReach, Seattle, USA

Kristin Beima-Sofie, Department of Global Health, University of Washington, Seattle, USA

Emily Lawrence, VillageReach, Seattle, USA

Corresponding Author

Bvudzai Magadzire

210 S Hudson St, Suite 307, Seattle, WA 98134

bvudzai.magadzire@villagereach.org

Word Count

4,972

For peer review only

ABSTRACT

Objective: Immunizations are highly impactful, cost-effective public health interventions. However, substantial gaps in complete vaccination coverage persist. We aimed to describe caregivers' immunization experiences and identify determinants of vaccine dropout.

Design: We used a community-based participatory research approach employing Photovoice, SMS exchanges, and in-depth interviews. A team-based approach was used for thematic analysis. The Increasing Vaccination Model guided the analysis and identification of vaccination facilitators and barriers.

Setting: This study was conducted in Zambézia Province, Mozambique, in Namarroi and Gilé Districts, where roughly 19% of children under 2 start but do not complete the recommended vaccination schedule.

Participants: Participants were identified through health facility vaccination records and included caregivers of children aged 25-34 months who were fully-vaccinated (n=10) and partially-vaccinated (n=22). We also collected data from 12 health workers responsible for delivering immunizations at the selected health facilities.

Results: Four main patterns of barriers leading to dropout emerged: 1) social norms and limited family support place the immunization burden on mothers; 2) perceived poor quality of health services reduces caregivers' trust in vaccination services; 3) concern about side effects causes vaccine hesitancy; and 4) caregivers hesitate to seek and advocate for vaccination due to power imbalances with health workers. COVID-19 created additional barriers related to social distancing, mask requirements, supply chain challenges, and disrupted o utreach services. For most caregivers, dropout becomes increasingly likely with compounding barriers. Caregivers of fully-vaccinated children noted facilitators, including accompaniment to health facilities or assistance caring for other children, which enabled them to complete vaccination.

Conclusions: Overcoming immunization barriers requires strengthening health systems, including improving logistics to avert vaccine stockouts and building health worker capacity, including empathic communication with caregivers. Consistent and reliable immunization outreach services could address access challenges and improve immunization uptake, particularly in distant communities.

Strengths and limitations of this study

- Photovoice methods allowed participants to visually share their stories and perspectives, generating rich, detailed, and nuanced information about the barriers that they experience in fully immunizing their children.
- The community-based participatory research approach allowed community members to engage throughout the research stages, ensuring that the findings and resulting recommendations are community-centered.
- Due to the contextual nature of vaccination systems and vaccination barriers, the results may have limited generalizability beyond similar settings and populations.
- The COVID-19 pandemic prevented us from convening any focus groups and may have affected the quality of our participatory analysis activities, which had to be conducted virtually rather than in-person.

For peer review only

INTRODUCTION

Childhood immunizations are highly impactful and cost-effective public health interventions, estimated to annually save 2-3 million lives worldwide.[8] In 1974, the World Health Organization (WHO) established the Expanded Program on Immunization (EPI) to guide the development and implementation of vaccination programs globally. Since then, coverage of the initial vaccines (protecting against diphtheria, tetanus, pertussis, measles, poliomyelitis, and tuberculosis) has increased from fewer than 5% of children in low- and middle-income countries (LMICs) to more than 85%.[9,10] However, significant gaps in vaccination coverage remain. In 2019, an estimated 14 million children were unimmunized, and 5.7 million were under-immunized,[1] predominantly from lower socioeconomic and rural populations in LMICs.[2] The COVID-19 pandemic has reversed recent progress, with an estimated 23 million children not completing basic vaccinations in 2020.[11]

The Mozambique EPI, launched in 1979, provides immunization services free of charge. Public sector health workers deliver EPI vaccines at fixed health posts, through routine mobile outreach activities, and sometimes during campaigns for specific vaccines.[12] Since the early 2000's, Mozambique has implemented several strategies, including the WHO/United Nations Children's Fund (UNICEF) Reaching Every District/Reaching Every Community (RED/REC) strategy, to improve vaccination outreach in remote communities.[12] According to the Mozambique EPI Manual, mobile vaccination brigades should provide services quarterly to communities residing further than eight kilometers from a health facility.[13] These outreach events are important for facilitating access to vaccines, as roughly 65% of the country's population lives in rural areas.[14] However, country-wide implementation of immunization outreach strategies has been impeded by insufficient funding and limited material and human resources, resulting in coverage gaps.[12]

Vaccination coverage in Mozambique increased dramatically from 47% in 1997 to 63% in 2003, but progress has since slowed; by 2015, only 66% of children were fully vaccinated. Within Mozambique, Zambézia Province has the lowest vaccination coverage; in 2015, 50% of children under 2 had received the first doses of recommended immunizations, and 38% had started but dropped out from the vaccination schedule.[3] Low coverage of the first set of recommended vaccines indicates poor access to services or lack of vaccination acceptance, while high dropout rates suggest health system failures to successfully deliver repeated doses.[15] Determinants of immunization dropout have been shown to be highly contextual, including combinations of individual, inter-personal, and health systems factors.[5] We conducted interviews with caregivers of young children and health workers, using a community-based participatory research (CBPR) approach, to identify key influences on under-2 immunization dropout in two districts in Zambézia Province, Mozambique.

METHODS

Study Design

We conducted a qualitative study using CBPR and photovoice methods. CBPR engages community representatives throughout the research process, reducing power imbalances between researchers and participants, creating an environment in which participants feel comfortable discussing sensitive topics, and facilitating the co-creation of contextually-sensitive and community-centered knowledge.[16] Four

For peer review only

Mozambican caregivers (referred to as Caregiver Researchers) were recruited from the local communities, were trained in research ethics and qualitative methods, led data collection, and assisted with data analysis.

The Increasing Vaccination Model, developed by the WHO Behavioral and Social Drivers of Vaccination (BeSD) working group,[17] guided data collection and analysis. We adapted this model to incorporate elements of the UNICEF Caregiver Journey Model, dividing the "practical issues" category into three sub-categories to capture the vaccination timespan (pre-, during-, and post-service) (Appendix A).

Study Setting, Population, and Recruitment

The study was conducted between February 2020 and March 2021 in Gilé and Namarroi Districts, Zambézia Province. Zambézia, the second-most populated province in Mozambique, has many hard-to-reach communities with some of the lowest health outcomes; a child born in Zambézia is twice as likely to die before age five than a child born in the capital city of Maputo.[12,18] Of the province's 16 districts, Namarroi and Gilé have the highest under-immunization rates, both at roughly 19%.[19] Gilé District has 11 fixed health facilities, serving a population of roughly 205,000.[19] Namarroi District has 10 fixed health facilities, serving a population of approximately 155,000.[19]

The study population included caregivers of fully- or partially-vaccinated children who lived in catchment areas of the selected health facilities, and health workers who administer immunizations at those facilities. 'Caregivers,' defined as parents or other guardians who take primary healthcare-seeking responsibility for the child, were eligible to participate if their child was 25-34 months old, an age by which the child should have received all routine childhood immunizations according to the Mozambique immunization schedule (Table 1). Caregivers were considered to be in the fully-vaccinated group (FV caregivers) if their child had received all 15 recommended immunizations, or the partially-vaccinated group (PV caregivers) if their child had received at least one but not all recommended immunizations. Health workers were eligible if they worked at the selected health facilities and if they were responsible for administering vaccinations to children under 2.

Table 1: Vaccination schedule and vaccination status of partially-vaccinated children

Vaccine	Dose	Age of administration per Mozambique EPI schedule		Partially-vaccinated children who received the dose (n=22)*
		Ideal	Allowed Range	
Tuberculosis	BCG	Birth	Birth – 23 months	22 (100%)
Oral Polio	OPV 0	Birth	Birth – 6 weeks	15 (68.2%)
	OPV 1	2 months	6 weeks – 23 months	19 (86.36%)
	OPV 2	3 months	10 weeks – 23 months	15 (68.2%)
	OPV 3	4 months	14 weeks – 23 months	12 (54.6%)
Pentavalent: Diphtheria, Pertussis, Tetanus, Hepatitis B, Haemophilus influenzae type B	DPT-HepB-Hib 1	2 months	6 weeks – 23 months	20 (90.1%)
	DPT-HepB-Hib 2	3 months	10 weeks – 23 months	15 (68.1%)
	DPT-HepB-Hib 3	4 months	14 weeks – 23 months	13 (59.1%)
Pneumococcal conjugate	PCV 1	2 months	6 weeks – 23 months	21 (95.45%)
	PCV 2	4 months	14 weeks – 23 months	17 (77.3%)

For peer review only

	PCV 3	9 months	9 months – 23 months	12 (54.6%)
Rotavirus	RV 1	2 months	6 weeks – 23 months	17 (77.3%)
	RV 2	3 months	10 weeks – 14 weeks	12 (54.6%)
Inactivated Polio	IPV	4 months	14 weeks – 23 months	7 (31.8%)
Measles, Rubella	MR 1	9 months	9 months – 23 months	3 (13.6%)
	MR 2**	18 months	18 months – 23 months	3 (13.6%)

*Note: Vaccination status is according to vaccination cards, or health facility records when no cards were available. Twins were counted as a single child.

**Note: The second dose of MR was added to the Mozambique EPI schedule in November 2017. Due to the timing of the study, completion of MR 2 was not considered when determining vaccination status.

We purposively selected eight health facilities (four per district) as recruitment sites to achieve a mixture of rural and peri-urban environments. Eligible caregivers were identified based on the facilities' immunization records. The Caregiver Researchers visited caregivers' homes with the aim of enrolling approximately 20 PV caregivers and 10 FV caregivers. During recruitment, we verified vaccination status via home-based vaccination cards, which were considered more accurate when there were discrepancies with health facility records. Sixteen caregivers from each district were enrolled (Figure 1), whereupon data saturation was reached. The study team purposively recruited and enrolled six health workers from each district, including at least one from each of the selected health facilities. No health workers declined to participate.

Data Collection

Caregiver Researchers were recruited via an open job posting advertised in Namarroi and Gilé Districts. Applicants were required to have a 24-36 month-old child, and women were prioritized, as they were more representative of the study population. Three women from each district were selected based on these requirements and on their level of education, fluency in local languages, and prior research experience. They participated in a five-day in-person training on qualitative research methods, and contracts were extended to the two women from each district with the best performance during the training.

Guides for caregiver and health care worker semi-structured interviews were developed in English based on the Increasing Vaccination Model and translated into Portuguese (Appendices B and C). The guides were piloted with the Caregiver Researchers and one health worker. Caregiver Researchers provided participants with study information. Basic demographic information was collected from all participants prior to each interview. Illiterate participants provided thumbprints instead of signatures, otherwise written consent was obtained, including for use of photographs of them and their children.

Photovoice and semi-structured interviews were used to document caregivers' experiences with the under-2 immunization process and to identify immunization completion barriers and facilitators. Photovoice, a method through which people can share their stories using photographs that visually represent their experiences and perspectives, can empower participants to craft rich stories, generating data that might not be captured through standard interview techniques.[20,21] Caregiver Researchers provided cameras to caregiver participants, gave detailed instructions on their use (Appendix D), and asked caregivers to take photos related to their experiences immunizing their child. Caregiver Researchers

For peer review only

returned after two to six days and conducted audio-recorded, in-depth interviews centered around the caregivers' photos. Initial interviews and transcripts were reviewed by EL and JP, who provided feedback and additional training to improve quality of subsequent interviews. Interviews were conducted in the local language of Elomué and lasted an average of 38 minutes. Following each interview, Caregiver Researchers completed written interview debriefs, noting key insights. Audio-recorded caregiver interviews were transcribed and translated from Elomué to Portuguese by Caregiver Researchers, audited for quality by study team members, and then translated into English by an external translator and ME.

SMS exchanges and semi-structured interviews were used to document health workers' perceptions of caregiver immunization experiences, their beliefs about causes of dropout, and their experiences administering vaccines to children under 2. SMS exchanges took place during January and February 2021. Each health worker was provided a phone with the Telegram application set up for group chats with the Caregiver Researchers and study team members. Over a three-week period, health workers were asked to send photos and messages whenever they had an experience or observation related to under-2 immunization. Caregiver Researchers replied with follow-up probes to collect additional information. If more than two days elapsed without any messages exchanged, the Caregiver Researchers sent follow-up reminders. On average, each health worker sent 33 messages (range 11-51 messages). In February 2021, Caregiver Researchers conducted audio-recorded interviews by phone with health workers in Portuguese, lasting an average of 70 minutes. Health worker interview audio recordings and SMS exchanges were transcribed and translated from Portuguese to English by an external translator.

Data Analysis

A team-based approach was used to perform a thematic analysis.[22] An initial codebook was developed deductively based on the Increasing Vaccination Model. Additional codes were added inductively following review of the first six transcripts, and the codebook was reviewed and updated by the Caregiver Researchers prior to coding. JP and EL individually coded the first three transcripts, and BM reviewed the coded transcripts for intercoder agreement. JP and EL then divided and independently coded the remaining transcripts in ATLAS.ti Version 9 and summarized the main findings by vaccination status.

Caregiver Researchers participated in a virtual two-week participatory analysis process facilitated by Portuguese-speaking study team members (AI, ME), in which they reviewed code summaries and representative quotations, identified themes and key barriers to vaccination, and drew comparisons between PV and FV caregivers. An online data collaboration platform (Miro, 2021) was used to facilitate collaboration and establish consensus on themes. The Photovoice photographs provided additional insight on what caregivers believed were the most significant aspects of their vaccination journeys. JP and EL reviewed the themes and photos to identify dropout patterns related to the Increasing Vaccination Model.

Patient and Public Involvement

The Caregiver Researchers, as representatives of the study population, participated in data collection and analysis. Study results were disseminated to most participants during follow-up workshops in April and May 2021. Participants will be involved in dissemination of results back to their communities and to local health authorities.

Ethics Statement

For peer review only

The Mozambique National Bioethics Committee for Health approved the study procedures (Ref:259/CNBS/20) and the University of Washington Institutional Review Board exempted the study from review (STUDY00011999).

RESULTS

Participants

Thirty-two caregivers participated in the study, and all were the mothers of the children. The majority lived in catchment areas of rural health facilities. On average, PV caregivers were older and less educated than FV caregivers (Table 2). Partially-vaccinated children were missing an average of 5.1 vaccines (range 1-14 missing vaccines), with the most commonly missed vaccines being Inactivated Polio and Measles-Rubella (Tables 1 and 2).

Table 2: Characteristics of caregiver participants and their children

	Partially-vaccinated (n=22) N (%) or median (IQR)	Fully-vaccinated (n=10) N (%) or median (IQR)
District		
Namarroi	11 (50%)	5 (50%)
Gilé	11 (50%)	5 (50%)
Health facility geography		
Rural	16 (73%)	7 (70%)
Peri-urban	6 (27%)	3 (30%)
Caregiver age (years)	26 (22-30)	21 (20-21.8)
Caregiver education		
Some primary	11 (50%)	3 (30%)
Completed primary	11 (50%)	7 (70%)
Child age (months)	30 (30-32)	31.5 (29.3-32)
Child sex		
Female	9 (41%)	6 (60%)
Number of missing vaccines (of 15 possible)	3.5 (2-8.8)	0

Twelve health workers participated in the study (Table 3). Most were male and had completed secondary education. Health workers had worked at the selected health facilities for an average of 4.7 years. All had children of their own, including five with children under 2.

Table 3: Characteristics of health worker participants

	N (%) or median (IQR)
District	
Namarroi	6 (50%)
Gilé	6 (50%)
Education Level	
Completed secondary	11 (92%)
Completed primary	1 (8%)

For peer review only

Work Experience	
Total years of work experience	7 (4.5-9.3)
Years at selected health facility	4 (2-5)
Job title	
Preventive Medical Technician	6 (50%)
General Nurse	4 (33%)
Maternal and Child Health Nurse	1 (8%)
Nutrition Technician	1 (8%)
Immunization trainings	
Attended immunization-specific training within past 5 years	5 (42%)
Attended immunization-specific training 5 or more years ago	2 (17%)
No formal trainings but met with colleagues to discuss introduction of new vaccines	2 (17%)
Never participated in vaccination trainings	3 (25%)
Sex	
Male	9 (75%)

Key Influences on Vaccine Dropout

Vaccination barriers arose in all categories of the Increasing Vaccination Model. In general, PV caregivers described abandoning vaccination after encountering multiple cross-domain barriers, rather than dropout being caused by a single barrier.

> *"…my father's illness until death, in addition to my illness, money and not even someone to help me, these are the events that happened during the first two years of my son's life." – PV Caregiver from Gilé*

Our analysis of caregiver experiences, coupled with insight from health workers, revealed four cross-domain patterns of barriers and facilitators to vaccination completion. These patterns related to, 1) social norms and limited family support; 2) perceived poor quality of health services; 3) concern about side effects; and 4) power imbalances between caregivers and health workers (Table 4). COVID-19 created additional barriers to vaccination completion.

Table 4: Cross-domain influences on vaccine dropout

Social norms and limited family support place the burden of vaccination on mothers, compounding the challenges of accessing vaccination services.
<u>Distance:</u> *"If it weren't for the distance, the children would be fully vaccinated, but we have to wait to sell the crop to have enough money and take the child to vaccination." – PV Caregiver from Gilé*<br><br><u>Physically Challenging:</u> *"If there is a possibility of a mobile brigade, with advance warning, I will take advantage of completing my daughter's missing vaccine, otherwise I have no alternative. Since walking to [the health facility] with this summer sun is very difficult." – PV Caregiver from Gilé*<br><br><u>Illness and Injury:</u> *"I came back from the hospital and sat here on the mat… My leg was in a lot of pain again on my way to the hospital and I ended up not vaccinating [my child]. I'm not going there anymore.… When I ask my husband to accompany our child to the hospital, he says that he is not able*

For peer review only

to take the child on his back.… But walking from here to the hospital is complicated with so much pain that I feel." – PV Caregiver from Namarroi<br><br>Family Support: *"The father has always helped me to remember the dates set to take the children to the hospital. He would wake me up early to bathe the children to go to vaccinate, and on the way back he would run to meet me to carry one of the children, because he knew that on the days that the children were vaccinated, they cried a lot." – PV Caregiver from Gilé*<br><br>Women's Role in Society: *"I was talking to the caregiver [who didn't complete vaccination], and she explained that she was sick and could not come to the health unit or to the mobile brigades, and we asked her about her husband. She explained that in the community, it is the woman who takes care of the children, and men are concerned with other things." – HW from Namarroi*
Perceived poor quality of health services reduces caregivers' trust in the health system.
Dissatisfaction: *"I am just lamenting very much, about the dosage of pills we are given when we go to the hospital; when we have travelled a long distance with a sick child to get half a pill, that is demotivating.… If we go to the hospital with malaria, they should give us malaria and paracetamol tablets with full dosages.... We would be satisfied despite the distance." – PV Caregiver from Gilé*<br><br>Stockouts: *"Sometimes when we arrived at the hospital, they claimed that there were no vaccines, so the child was just weighed…. When that happened, I'd regret the distance I had to travel.… The following month I would not return with fear of spending money and not finding the vaccine." – PV Caregiver from Gilé*<br><br>Human Resources: *"Many times there is lack of human resources.… So it is a challenge, it is a major effort that I have made in order to satisfy everyone who came to seek our services." – HW from Namarroi*
Concern about side effects, exacerbated by "accumulation" of vaccines, leads to hesitancy.
Fear of Vaccine "Accumulation": *"[Caregivers] know that when a mother missed vaccinations, the child will receive more vaccines to cover those vaccines that are missing. No mother likes to see her child being given 3 or more vaccines; it is painful for the child." – HW from Gilé*<br><br>Lack of Information: *"The child caught fevers [after vaccination], so I'd leave her lying down to rest and I sat beside her to calm her down. Sometimes she had two or three days with fevers and swollen legs. She got more fevers in the months that I had multiple vaccines at the same time. As I was not informed about the side effects of getting different vaccines at the same time, the child would get home with many fevers and worry me." – PV Caregiver from Gilé*<br><br>Perception of Normal Side-effects: *"The vaccine is important.… Even with the fevers and swelling the child gets after a vaccine, we cannot give up. It will pass and the child will continue playing." – PV Caregiver from Gilé*
Power dynamics at the health facility make caregivers hesitant to seek or advocate for vaccination.
Out-of-Schedule: *"We should take the child to the hospital [on the scheduled dates] for vaccinations to avoid being yelled at, because all mothers who do not take their children on the scheduled dates are yelled at." – FV Caregiver from Gilé*

For peer review only

Importance of Bathing: *"Whenever I take my child to the hospital to vaccinate, I first bathe to avoid rejection and other illnesses that can be caught due to poor hygiene. Since when you take the child without bathing, it will not be vaccinated, it is refused.… When taking the child without bathing, I am afraid that other mothers will laugh and consider me as a dirty person." – PV Caregiver from Gilé*

Vaccination Cards: *"Some mothers, when they lose or get the child's health card wet, they get afraid of returning to the health facility to continue with the child's vaccinations." – HW from Namarroi*

Vaccination Session Hours: *"There are mothers who live far away and do not have motorbikes or bicycles, and are therefore coming on foot to the health facility. There are also mothers who prefer to arrive later to avoid the floods that occur in the morning.… However, if the mother arrives after 3PM, it will no longer be possible [to vaccinate] because the technician has already finished his activities.… This is because the opening of a refrigerator has rules, and it should be opened only twice a day, in the morning and in the afternoon." – HW from Gilé*

Abbreviations: partially-vaccinated (PV), fully-vaccinated (FV), health worker (HW)

Social Norms and Limited Family Support Place the Burden of Vaccination on Mothers, Compounding the Challenges of Accessing Vaccination Services

For many caregivers, traveling to a health facility required significant investments of time, effort, and money. Some caregivers described selling crops or buying less food to save money for transport to the health facility. When they had no money for transportation, caregivers described having to walk for several hours while carrying their children, often through challenging and dangerous terrain and inclement weather, even spending the night because the trip was too long to complete in a single day.

> *"The hospital is very distant. On the days we have to go on foot, we come home with our feet swollen from so much walking. There are times when you cannot go home because it is late, and you are forced to sleep there, even with nowhere to stay." – PV Caregiver from Gilé*

PV caregivers noted that given the long distances required to travel to receive vaccinations, lack of support from other family members was a major barrier to completing the vaccination schedule. Limited family support was in part due to community beliefs that vaccination responsibility resides with mothers and that fathers should not be involved in the process. This belief resulted in some mothers abandoning the process when they were unable to travel to the facility due to illness or injury, and their husbands and other family members did not help bring the children to the health facility. Several other mothers reported dropping out due to lack of support with managing other conflicting obligations, such as caring for other children.

> *"[My aunt] had a toothache and couldn't stay with [my other children]. When she told me this, I was left with no way out; I couldn't go to the hospital and leave my children alone." – PV Caregiver from Gilé*

Social support from family and friends helped FV caregivers to overcome vaccination barriers. FV caregivers described receiving support in a variety of ways including reminders from family members, money for transport to the health facility, accompaniment to the facility by other caregivers or a family member, help with other household responsibilities, and support in taking the child to the facility when the mother is sick.

For peer review only

Both caregivers and health workers noted that reliable, consistent mobile brigades would help to address the practical barriers associated with traveling to health facilities, especially for those mothers who lack support from family members. Several caregivers mentioned that mobile brigades had previously enabled them to complete vaccinations of older children, but that the brigades had become infrequent and unreliable. Health workers described barriers to running mobile brigades, including insufficient fuel and lack of staffing to manage both fixed posts and outreach services.

> *"We need motorcycles and fuel so that we can run mobile brigades in the communities.... There are times when this doesn't happen 100% because the bikes are damaged, and we can't do maintenance.... When we do not have funds for fuel, sometimes the mobile brigades are paralyzed." – HW from Namarroi*

Perceived Poor Quality of Health Services Reduces Caregivers' Trust in the Health System

Many caregivers began the vaccination process highly motivated, and some described having positive experiences at health facilities. However, other caregivers were dissatisfied with their experiences at the service delivery point due to vaccine stockouts, long wait times, and inconsistent health facility hours. PV caregivers also described being frustrated by interactions with disengaged health workers. After expending significant effort to reach the health facility, some caregivers arrived to find that health workers were late or were busy talking on their phones, causing them to lose faith in the health system and lose motivation to vaccinate.

> *"I arrived at the hospital at a good time, before the activities started. I was waiting for the nurses until the time they arrived.... I was very indignant, since the nurses attended a person and interrupted to answer telephone calls that lasted many hours without consideration for mothers who live very far from the hospital." – PV Caregiver from Gilé*

These dissatisfying experiences extended to general health services too; several caregivers discussed visits for treatment of malaria or other illnesses in which they received only partial doses of medications, leading them to also question the quality of vaccination services and abandon the vaccination process, rather than continue to invest time, effort, and money.

Health workers described their own challenges in delivering vaccines and providing quality care to every child, including stockouts of vaccines and other supplies and high work burden due to insufficient human resources. Many health workers described vaccine stockouts lasting weeks to months with no guarantee of when doses would be available again. Several health workers raised concerns that aligned with those expressed by caregivers: that caregivers would not return after encountering stockouts due to distances and other difficulties associated with vaccine care seeking.

> *"The whole province... has been out of stock of [the polio vaccine] for 2 months.... Considering that the vaccination program has a certain calendar, it is always embarrassing that this happens, because the children who should have had vaccinations in that period do not get them." – HW from Gilé*

Concern about Side Effects, Exacerbated by "Accumulation" of Vaccines, Leads to Hesitancy

Some caregivers understood that side effects such as swelling at the injection site or fever are common and should not be a reason to stop vaccination. However, others expressed concerns about these

For peer review only

reactions, especially when they lasted for several days. This concern was also greater for caregivers who lived far away and felt that the long walk home aggravated the side effects. Some caregivers worried that "simultaneous" or "accumulated" vaccines – those given to catch up on missed doses -- would exacerbate the fevers and swelling, causing some to drop out of the vaccination process.

> *"…what made me abandon it were the side effects of the vaccine. I wanted to protect my son by abandoning the service and I didn't know I was harming the child.... On the days that he received the vaccines simultaneously, he would have many fevers. This happened when he spent many months without vaccinating." – PV Caregiver from Gilé*

Health workers recognized these concerns. They felt that caregivers might not be aware that reactions are relatively common and are rarely harmful in the long-term, and they noted that better communication with caregivers is important for reducing dropouts caused by fear of side effects.

> *"[The health worker] needs to sensitize the mother…why the child is getting that vaccine, and what will be the effect of that vaccine. For example, if the child has a fever after that vaccination, you tell the mother not to worry because it is normal..." – HW from Namarroi*

Power Dynamics at the Health Facility Make Caregivers Hesitant to Seek and Advocate for Vaccination Services

Several caregivers described negative interactions with health workers. Caregivers feared that health workers would scold them and send them home if they came out of schedule after missed doses, did not bathe their child prior to the visit, or forgot to bring their vaccination card.

> *"If you have too many absences, when you arrive at the hospital you are insulted for not taking the child for the vaccination, even though you try to explain that it was not of your own will but because of the distance." – PV Caregiver from Gilé*

There were strong social norms around bathing the child before vaccination to avoid being rejected or humiliated. Caregivers' fear of humiliation was exacerbated by the public nature of the health facility, where immunization activities and conversations take place in front of other caregivers due to lack of private rooms.

Caregivers frequently discussed the importance of vaccination cards. Mothers who had given birth outside of a facility often lacked vaccination cards and feared that if they requested one, health workers would admonish them for the non-institutional delivery. Caregivers also feared being yelled at and rejected by health workers over lost or damaged cards.

> *"[My child] didn't get all the vaccinations because she didn't have a card. When it got torn, I arrived at the hospital to ask for a new card. I was told to get the previous one and I was left with no options." – PV Caregiver from Namarroi*

Caregivers noted that power dynamics sometimes also resulted in missed vaccination opportunities. A few mothers described instances when health workers sent them home without delivering any vaccinations. Even when caregivers believed that their children needed to be vaccinated, they were hesitant to approach the health workers to request the vaccine, fearing that they would be yelled at.

For peer review only

"Many times when I went to the hospital, they just sent my daughters to weigh and said they had already completed the vaccinations and didn't even check the children's cards. But as a mother, I knew that the last vaccine was missing and I couldn't question it for fear of being humiliated." – PV Caregiver from Gilé

From the health worker perspective, however, some of these missed opportunities were due to challenges that they faced regarding vaccine delivery and cold chain policies. Some health workers said they do not vaccinate children over the age of two, and one described policies that limit them to opening vaccine freezers just once at the start of the vaccination session and once at the end. If caregivers arrived after the vaccines were put away, the health workers could not retrieve the vaccines. Additional missed opportunities to vaccinate occurred at two health facilities; at one, there was no maternal and child health nurse to manage vaccination of newborns on weekends, and at the other, the maternal and child health nurses had been trained in vaccination but were not administering them to newborns.

"All deliveries that take place on the weekends must return on Monday to vaccinate. Sometimes there are missed opportunities because mothers live far away.... [Maternal and child health] nurses have training in vaccination, and it is possible to manage the vaccination of newborns - just apply the BCG vaccine and then dismiss the mothers - but this is not happening." – HW from Gilé

The data suggest a breakdown in communication between health workers and caregivers; health workers do not always communicate their logistical and resource constraints, and caregivers are afraid to ask about why their children are not receiving vaccines.

"For a few months that I went to the hospital they just weighed the children and returned the card to me without saying anything, but I was afraid to ask. I was silent without knowing if it was a lack of vaccines at the hospital or bad luck for me." – PV Caregiver from Gilé

Impacts of COVID-19 on Vaccination Experiences

Though some caregivers and health workers felt that COVID-19 had not impacted the vaccination process, others described changes to health services and other barriers to vaccination resulting from the pandemic. In some cases, caregivers were hesitant to go to the health facility due to fear of contracting COVID-19 or general discomfort around the new health facility policies. Mask requirements were another reported deterrent, as caregivers who did not have their own masks knew that they would not be served.

"In the hospital, without the use of masks, people are not attended, as well as on public transport. Our habits of shaking hands and staying close to our friends is also forbidden. These are things that make us distressed and worried without knowing how long it will take before it passes." – PV Caregiver from Gilé

At the health facility level, health workers described shortages of general supplies, such as gloves, and increased vaccine stockouts.

"At times, we lacked some vaccines because…the borders closed…due to COVID-19.… For example, we are currently without BCG and polio vaccines.… There is difficulty in [transporting the vaccines to] the province." – HW from Namarroi

For peer review only - http://bmjopen.bmj.com/site/about/guidelines.xhtml

Health workers also mentioned challenges maintaining social distancing because the waiting rooms are too small to accommodate all the caregivers, forcing caregivers and their children to wait outside even in hot sun or rain.

Both caregivers and health workers noted that mobile brigades have been especially infrequent during the COVID-19 pandemic. Health workers described difficulties in organizing mobile brigades due to social distancing requirements as well as lack of fuel and required supplies. Caregivers commented that they were unaware of brigade schedules due to closures of schools and churches, which previously were their main sources of information about brigades. When mobile brigades did happen, health workers reported that far fewer caregivers than usual brought their children due to fears of congregating in groups.

> *"Now we go to the community, and we find there are not enough children due to the pandemic, and messages are arriving in the communities saying that we cannot concentrate…fifty people together…. If before COVID-19, I could reach 200 children in immunization, today when I go, I can only reach 30 to 50 children." – HW from Namarroi*

DISCUSSION

Our study highlighted the immense effort that caregivers put into vaccinating their children and the numerous and difficult barriers they face. Primary barriers identified in our study included: practical issues related to health facility access, unreliable and poorly perceived service quality, negative interactions with health workers, caregiver lack of awareness of vaccine side effects, and unsupportive household dynamics. Our findings are consistent with many of the dropout determinants that have previously been identified in systematic reviews, including: access, health worker availability, missed opportunities, service reliability, family dynamics, lack of motivation, fear of side-effects, mistrust of the health system, and health staff attitudes and behavior.[4,5] Our findings also align with results from cross-sectional studies in Mozambique, which found that dropout was related to lack of access to vaccination facilities, household decision-making processes, and children born at home or outside of Mozambique.[6,7] Additional barriers that have been previously identified, but which did not arise explicitly, were maternal education and poverty. We did see, however, that illiteracy and cost were barriers, which relate to maternal education and poverty respectively.

Our study generated several new findings related to caregivers' interactions with health workers and their perceptions of service quality, caregivers' hesitancy related to vaccine "accumulation," the importance of family support, and the shortcomings of the existing outreach services. Fear of humiliation or reprimand contributed to caregivers' perceptions of poor service and discouraged them from continuing with vaccination once their child had fallen off schedule. The perceived poor quality of *general* health services influenced some caregivers' decisions to stop seeking vaccination services. Communication breakdowns may have made caregivers unaware of health workers' constraints, exacerbating their perceptions of poor service quality. Many caregivers in our study were more fearful of side effects when they believed that their child was receiving too many vaccines simultaneously and especially in the context of catch-up administration. Our findings showed that family support of mothers is a strong enabler to overcoming many vaccination barriers, but the dominant social norms exempt husbands and other family members from assisting with the vaccination process. Finally, the data showed that currently, outreach services are not meeting the needs of caregivers in distant

For peer review only

communities; mobile vaccination brigades have been infrequent, especially due to COVID-19, and caregivers do not always receive advance notice of them. Overall, vaccine dropout was related to compounding barriers that eventually tip the scales in favor of abandoning the vaccination cycle. This study has generated a more comprehensive understanding of how those barriers interact together.

Implications on Policy and Practice

Health systems strengthening is receiving increasing attention globally.[23] Our findings support the need for this approach to increase vaccination completion, including improved supply chains to ensure adequate stocks of vaccines and other supplies, sufficient health workforce, and health facilities that allow for privacy and confidentiality. Capacity building of health workers, including training, retraining, and supportive supervision is also needed.[24] These capacity building approaches should not only focus on technical content, but should support health workers to engage in respectful, empathic, and patient-centered communication.[25] Specifically, better messaging is needed on vaccine availability, scheduling and hours of immunization sessions, common reactions especially for off-schedule vaccines, and policies around issuing new vaccination cards. Involvement of community leaders, religious leaders, and community health workers in those communication strategies may improve vaccination uptake.[26] Ultimately, however, enhanced relationships with patients and the community also require a sufficient and motivated health workforce.[25]

Our findings suggest that re-investment in community-centered vaccine outreach services could improve completion rates. Regularly scheduled outreach events have the potential to greatly facilitate access to immunization services in distant communities, especially when communities are involved in planning them.[27] The WHO and UNICEF have endorsed the RED/REC strategy since 2002, [27,28] but our findings suggest that there has been inadequate resource allocation to implement RED/REC in the study communities.

To successfully implement these recommendations, there is a need for further research on the best channels of communication between health facilities and the communities they serve, as well as on how best to design and implement empathy-building trainings for health workers. Additionally, there is a need to better understand how to engage husbands and other family members in the vaccination process to reduce the burden on mothers.

Our study has several limitations. The specific barriers we identified may not be generalizable to other contexts because influences on vaccine dropout are highly contextual, and determinants relate to social norms, health service policies, and geography. However, the complex nature of compounding barriers may translate to other settings. An additional limitation is that this study was conducted during the COVID-19 pandemic, when many health services were disrupted. While this provided a unique opportunity to examine barriers caused by this crisis, this also may have biased our findings about dropout during non-pandemic times and may have affected the quality of the participatory analysis process, which was conducted virtually. Finally, during recruitment, 13 caregivers were not enrolled due to absence or seasonal relocation to their farms. These caregivers were disproportionately caregivers of PV children, and failure to include them may have influenced the results. Despite these limitations, this study demonstrates that immunization dropout is complex, with caregivers facing different combinations of barriers and facilitators. The findings highlight the need for improved quality and reliability of vaccination services and for community-centered solutions that remove practical barriers and empower caregivers to complete the vaccination process.

For peer review only

Author Contributions

J.P., B.M., A.D., B.C., J.C., and E.L. contributed to study design and logistics. A.D., A.I., B.B., L.J., and Z.M. contributed to data collection. J.P., B.M, D.D., A.I., B.B., L.J., Z.M., M.E., K.B., and E.L. contributed to data analysis. J.P. took the lead in writing the manuscript, with consultation from D.D., K.B., and E.L. All authors provided critical feedback and helped shape the final manuscript.

Acknowledgements

Samira dos Santos assisted with data collection as a Caregiver Researcher.

Competing Interests

None declared.

Funding

This work was supported by Wellcome Trust grant number 219237/Z/19/Z. The funder did not have a role in the study design, analysis, or data interpretation, or manuscript development.

Data sharing statement: No additional data is available.

Figures

Figure 1: Recruitment and enrollment of caregiver participants

For peer review only

REFERENCES

1 World Health Organization. Immunization coverage. World Health Organization 2020. https://www.who.int/news-room/fact-sheets/detail/immunization-coverage (accessed 23 Dec 2020).

2 Restrepo-Méndez MC, Barros AJ, Wong KL, *et al.* Inequalities in full immunization coverage: trends in low- and middle-income countries. *Bull World Health Organ* 2016;**94**:794-805B. doi:10.2471/BLT.15.162172

3 Ministério da Saúde- Misau, Instituto Nacional de Estatística- Ine, ICF. Inquérito de Indicadores de Imunização, Malária e HIV/SIDA em Moçambique (IMASIDA) 2015. Published Online First: 1 February 2018.https://dhsprogram.com/publications/publication-AIS12-AIS-Final-Reports.cfm (accessed 24 Feb 2020).

4 Rainey JJ, Watkins M, Ryman TK, *et al.* Reasons related to non-vaccination and under-vaccination of children in low and middle income countries: Findings from a systematic review of the published literature, 1999–2009. *Vaccine* 2011;**29**:8215–21. doi:10.1016/j.vaccine.2011.08.096

5 Favin M, Steinglass R, Fields R, *et al.* Why children are not vaccinated: a review of the grey literature. *Int Health* 2012;**4**:229–38. doi:10.1016/j.inhe.2012.07.004

6 Jani JV, De Schacht C, Jani IV, *et al.* Risk factors for incomplete vaccination and missed opportunity for immunization in rural Mozambique. *BMC Public Health* 2008;**8**:161. doi:10.1186/1471-2458-8-161

7 Shemwell SA, Peratikos MB, González-Calvo L, *et al.* Determinants of full vaccination status in children aged 12–23 months in Gurùé and Milange districts, Mozambique: results of a population-based cross-sectional survey. *Int Health* Published Online First: July 2017. doi:10.1093/inthealth/ihx020

8 UNICEF. Vaccination and Immunization Statistics. 2020.https://data.unicef.org/topic/child-health/immunization/ (accessed 30 Nov 2020).

9 Keja K, Chan C, Hayden G, *et al.* Expanded programme on immunization. *World Health Stat Q Rapp Trimest Stat Sanit Mond* 1988;**41**:59–63.

10 Peck M, Gacic-Dobo M, Diallo MS, *et al.* Global Routine Vaccination Coverage, 2018. *Morb Mortal Wkly Rep* 2019;**68**:937–42. doi:10.15585/mmwr.mm6842a1

11 World Health Organization. COVID-19 pandemic leads to major backsliding on childhood vaccinations, new WHO, UNICEF data shows. World Health Organ. News. 2021.https://www.who.int/news/item/15-07-2021-covid-19-pandemic-leads-to-major-backsliding-on-childhood-vaccinations-new-who-unicef-data-shows (accessed 17 Aug 2021).

12 Ministry of Health. National Immunization Program Comprehensive Multi-Year Plan (cMYP) 2015-2019. Republic of Mozambique 2014.

For peer review only - http://bmjopen.bmj.com/site/about/guidelines.xhtml

13 MISAU. Manual do Programa Alargado de Vacinação. Maputo, Moçambique: : República de Moçambique Ministério da Saúde 2019.

14 Instituto Nacional de Estatistica. INE Destaques. Inst. Nac. Estat. 2021.http://www.ine.gov.mz/ (accessed 28 Jun 2021).

15 Ghosh A, Laxminarayan R. Demand- and supply-side determinants of diphtheria-pertussis-tetanus nonvaccination and dropout in rural India. *Vaccine* 2017;**35**:1087–93. doi:10.1016/j.vaccine.2016.12.024

16 Leung MW, Yen IH, Minkler M. Community based participatory research: a promising approach for increasing epidemiology's relevance in the 21st century. *Int J Epidemiol* 2004;**33**:499–506. doi:10.1093/ije/dyh010

17 World Health Organization. Acceptance and demand. Essent. Program Immun. https://www.who.int/teams/immunization-vaccines-and-biologicals/diseases/tick-borne-encephalitis/essential-programme-on-immunization (accessed 13 May 2021).

18 Government of Zambezia Province. The Province. Portal Gov. Zambezia Prov. 2018.https://www.zambezia.gov.mz/por/A-Provincia (accessed 2 Jun 2021).

19 DPS Zambezia. Relatório anual de 2019 do Programa Alargado de Vacinação. Direcção Provincial de Saúde de Zambézia 2019.

20 Wang C, Burris MA. Photovoice: Concept, Methodology, and Use for Participatory Needs Assessment. *Health Educ Behav* 1997;**24**:369–87. doi:10.1177/109019819702400309

21 Milne E-J, Muir R. Photovoice: A Critical Introduction. In: *The SAGE Handbook of Visual Research Methods*. 1 Oliver's Yard, 55 City Road London EC1Y 1SP: : SAGE Publications, Inc. 2020. 282–96. doi:10.4135/9781526417015.n17

22 Guest G, MacQueen KM, Namey EE. *Applied Thematic Analysis*. SAGE Publications 2011.

23 Hafner T, Shiffman J. The emergence of global attention to health systems strengthening. *Health Policy Plan* 2013;**28**:41–50. doi:10.1093/heapol/czs023

24 Rowe AK, Rowe SY, Peters DH, *et al.* Effectiveness of strategies to improve health-care provider practices in low-income and middle-income countries: a systematic review. *Lancet Glob Health* 2018;**6**:e1163–75. doi:10.1016/S2214-109X(18)30398-X

25 Man JD, Mayega RW, Sarkar N, *et al.* Patient-Centered Care and People-Centered Health Systems in Sub-Saharan Africa: Why So Little of Something So Badly Needed? *Int J Pers Centered Med* 2016;**6**:162–73. doi:10.5750/ijpcm.v6i3.591

26 Goldstein S, MacDonald NE, Guirguis S. Health communication and vaccine hesitancy. *Vaccine* 2015;**33**:4212–4. doi:10.1016/j.vaccine.2015.04.042

27 World Health Organization. Reaching Every District (RED) A guide to increasing coverage and equity in all communities in the African Region. World Health Organization 2017.

For peer review only

28 Mantel C, Cherian T. New immunization strategies: adapting to global challenges. *Bundesgesundheitsblatt Gesundheitsforschung Gesundheitsschutz* 2020;**63**:25–31. doi:10.1007/s00103-019-03066-x

For peer review only

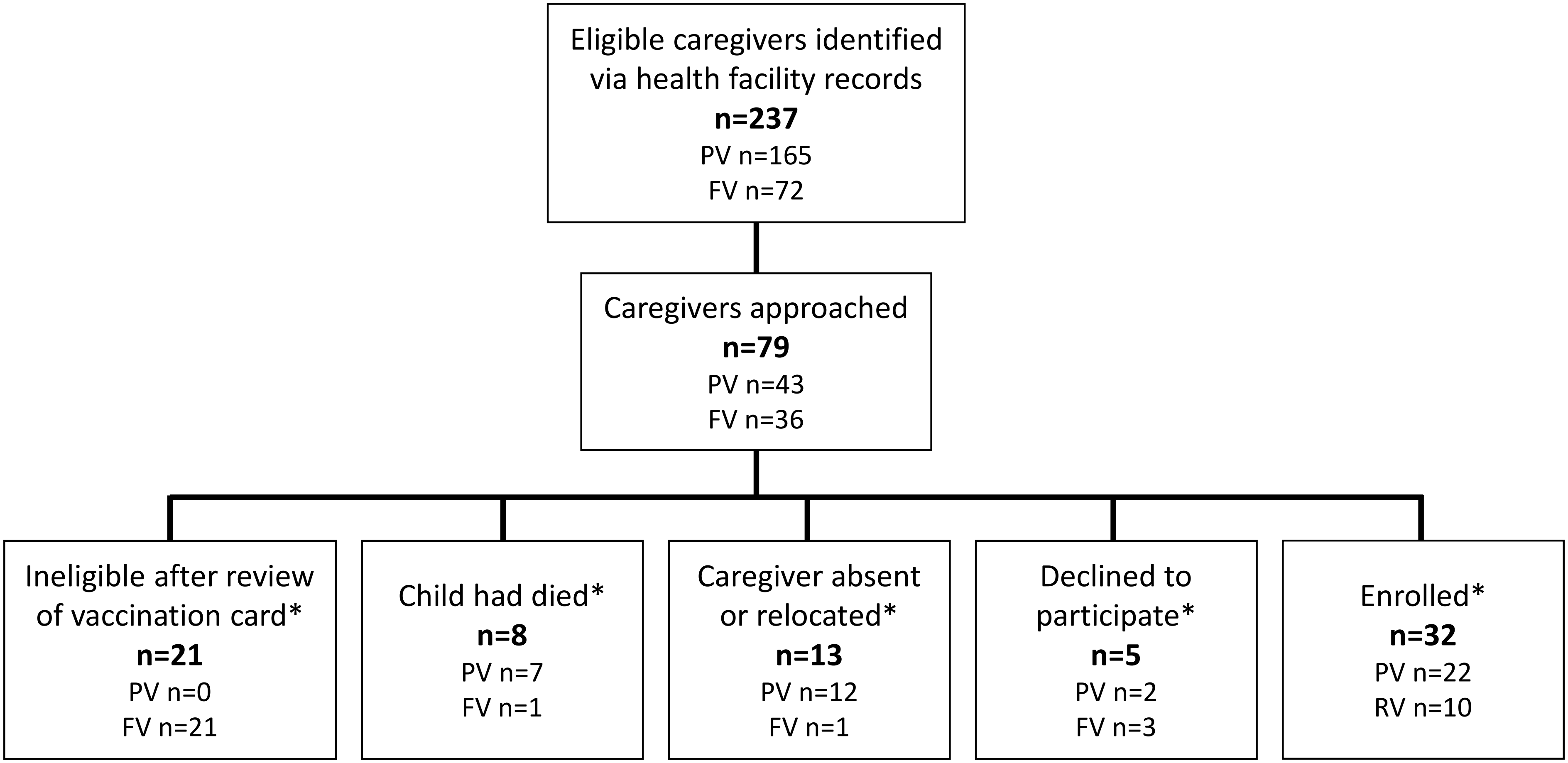

*Vaccination status reflects immunization card, when available

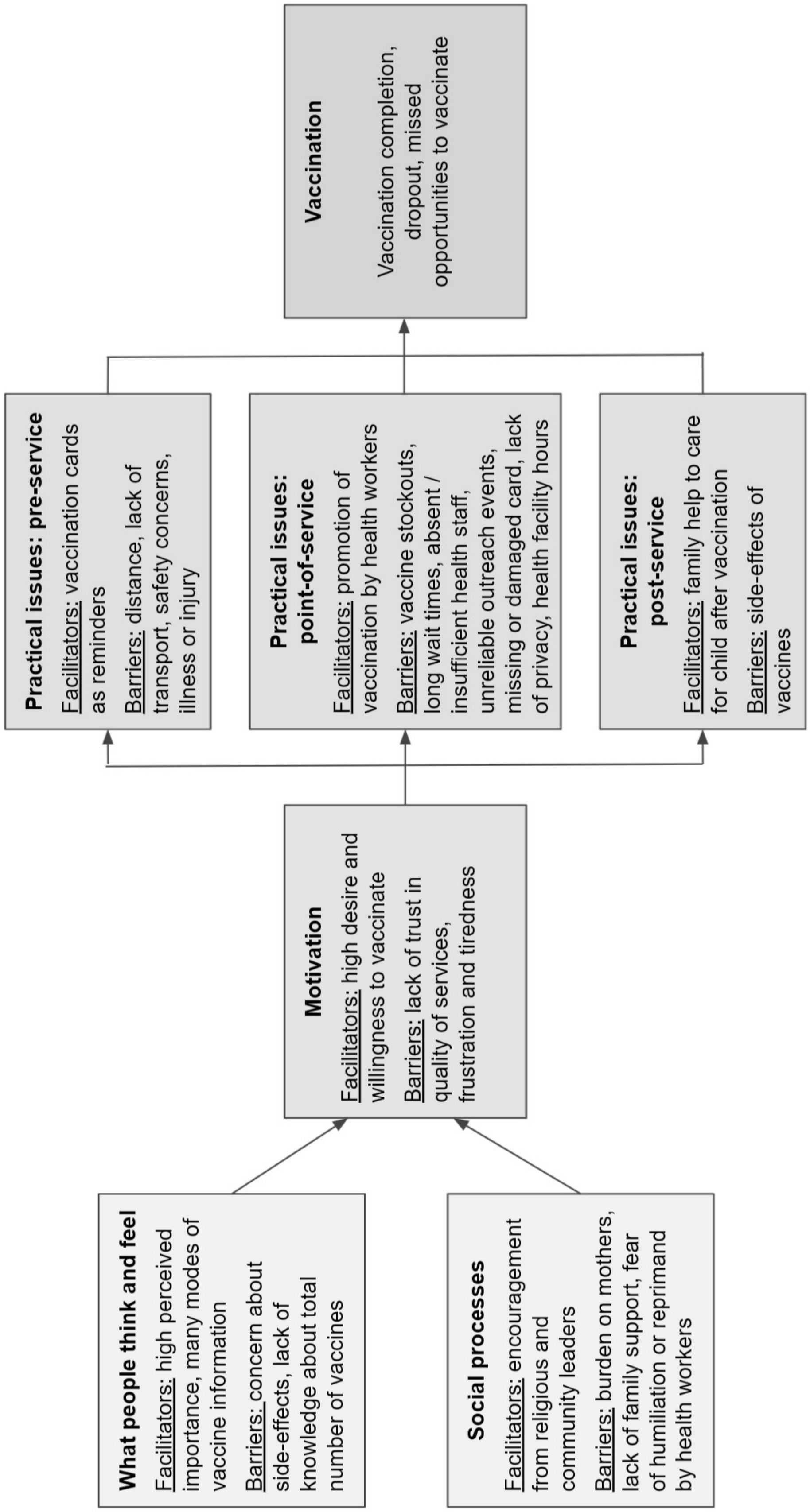

Appendix A: Adapted Increasing Vaccination Model

Appendix B: Semi-Structured Interview Guide for Caregiver Interviews

Introduction: Good morning/afternoon. I am back to go over the photographs and conduct the remainder of the interview with you. Is now still a good time?

Introduction to in-depth interview:

- This interview will last about 1.5 hours.
- Please know that what you say to us is confidential. We know how important this is to you. We also ask the other participants keep our discussions confidential.
- We would like to record the conversation so that we can document all the information you provide.
- To better understand immunization in your community, it is really important that you share your true beliefs and attitudes towards vaccination, as well as your personal experiences in vaccination of your children.

Informed consent for photos and recording the interview: Now before we get started, I would like to request your informed consent again to review the photographs and record the interview. As mentioned last week, your participation is voluntary and there is no penalty for not taking part. You may refuse to answer any question or withdraw from the study at any time.
Do you have any questions or would you like me to read the informed consent again?

If agree, have them sign the lines on the consent forms for the photographs and recording of the interview!

Discuss "Ground Rules" for the In-depth Interview

- Please don't answer your cell-phone during this interview.
- Do you have any other questions before we get started?

Review of Photovoice:

- Ask them to show the photos they have taken on the camera
- Ask them to pick 3-5 photos that best represent their experience of immunizing [child's name].
- Make sure to pick photographs that are clear!
- **Note: If there are any photos of them, their child or family members, request a signed photo release form by each individual in the photo! If it is the child, they can sign for their child.**
- Upload the selected photos to the computer and paste them in PowerPoint and begin the photovoice session and interview.
  - If the computer isn't working, review the photos on the digital camera and note down the photo number for future reference
- Turn on the recorder!
- Begin the interview using the interview guide

For peer review only

Semi-Structured Discussion Guide for Photovoice In-depth Interview

Instructions: To elicit rich descriptions from participants, when asking follow-up questions or encouraging them to talk more or give you more examples/details, use the following probes:

- *What do you mean by ____ ?*
- *Tell me more about ____.*
- *Can you give me an example of ____ ?*
- *Can you tell me about a time when ____ ?*
- *Can you tell me about the last time ____ ?*
- *Is there anything that made ____ easier?*
- *Is there anything that made ____ harder?*
- *(Can you tell me) Who ____?*
- *(Can you tell me) When ____ ?*
- *(Can you tell me) Where ____ ?*

Probes are completed using only verbatim participant words or phrases.

Interview Guide:

Before we discuss the photos more, can you tell us about how the photovoice process went for you? Did you discuss the task with anyone else in your family or community or have any help in taking the photos?

If yes, Did they have any influence on any of the photos you took?

- If so, in what way?
- Can you provide an example of how they impacted the photos you took and selected?

Once photos are selected, ***ask for each photo:***

- What do we see in this photo?
- What does this represent to you?
- How does this photo represent your vaccination journey?

Now, based on our activity with the photos, we have learned more about your experience, thank you. Now we have some more questions to better understand your experience immunizing your child and the things that influenced [child's name] immunization status. Do you have your child's immunization card which we can refer to during this interview?

1) To start, can you tell me about how people in your community talk about immunizations?
   a) How does this relate to how you feel about immunizing [child's name]?
   b) Has this changed at all with COVID-19?
      i) If yes, why and how?
      ii) If no, why not?

2) Can you describe to me what the experience was like getting immunizations for [child's name]?
   a) How many times did [child's name] get immunizations before the age of two? (look at immunization card)?

For peer review only

b) When did they get these immunizations?
   i) [if they got immunizations more than once] Were these experiences the same? Different?
   ii) How so?
c) Where did [child's name] get their immunizations?
   i) Did they get them all there?
   ii) What did you think about getting the immunizations there?
d) Who immunized [child's name]?
   i) How do you feel about that?
   ii) Did you trust the [health worker/nurse/doctor] who gave the immunizations?
      (1) Why or why not?
e) How satisfied were you with the experience(s) of immunizing [child's name]?
   i) What lead to you feeling this satisfied/dissatisfied?

3) When deciding to immunize [child's name], what things did you consider?
   a) Was there anyone who helped you make that decision?
      i) Who? How did they help?
   b) Did you intend for your child to be fully vaccinated according to the recommended schedule?
      i) If yes, why?
      ii) If no, why not?
   c) Did you have questions about the recommendations for immunizing [child's name]?
      i) Why or why not?
   d) How important did you feel getting these immunizations was, if at all, for [child's name]?
   e) Have things changed since COVID-19?
      i) If so, how?

4) What kind of information, if at all, did you receive about immunizing your child or about immunizations in general?
   a) When did you receive this information?
   b) Where did this information come from?
      i) Health center?
      ii) Social media (Facebook, WhatsApp)?
      iii) Neighbours?
   c) How often, if at all, did you receive information?
   d) To what extent did you trust this information?
   e) Did you get information about how many immunizations your child needed and when they were needed?
   f) Did this change with COVID-19? How?

For peer review only

5) [for those who have a child who dropped out] Can you describe why [child's name] didn't receive all their vaccinations by age two?
    a) What factors do you think contributed to that?
        i) Probe on:
            (1) What kind of information did you receive about your child's immunization schedule, if at all?
            (2) Did you know what your child's immunization schedule was?
            (3) COVID-19
    b) What was hard about trying to immunize [child's name]?
        i) Can you provide an example?
        ii) How, if at all, has this changed since COVID-19?
    c) What was easy about trying to immunize [child's name]?
        i) Can you provide an example?
        ii) How, if at all, has this changed since COVID-19?

6) [for those who have a fully-immunized child] What factors do you think contributed to being able to fully immunize [child's name] by the age of two?
    a) What was hard about trying to immunize [child's name]?
        i) Can you provide an example?
        ii) How, if at all, has this changed since COVID-19?
    b) What was easy about trying to immunize [child's name]?
        i) Can you provide an example?
        ii) How, if at all, has this changed since COVID-19?

7) How do you wish the experience of getting all the recommended immunizations for [child's name] could be easier, if at all?
    a) What would you change?
    b) What would you keep the same?
    c) How would these changes look in times where there is no COVID-19?
    d) How does it look now with COVID-19?

Conclusion: Are there any final thoughts you wish to share about your experiences with immunizing your 2-3 year old child?

We would like to thank you for taking the time to discuss this important topic with us!

For peer review only

Appendix C: Semi-Structured Interview Guide for Health Worker Interviews

Introduction to Structured Phone Interview: The purpose of the debrief activities is to reflect on and discuss your messages with the healthcare workers you corresponded with. You will use the message notes to debrief with the healthcare workers about the immunization process. This is a rough guide of questions to ask the healthcare workers, and additional questions or topics can be discussed based on specific observations that you made from the messages.

Interview Questions

1) Now, I am going to walk through some of the activities you mentioned in your messages, and I would like to get your perspective on:
    a) Were these typical [experiences, processes, observations]?
        i) If so, how? If not, how did they vary from normal?
    b) Is there anything else you would like to share or explain about the [experience, process, observation] that may not have been captured in the messages?

2) How do you see your role in immunizations?
    a) How do you feel about that role?
    b) Do you feel supported in your role? If not, why? If yes, how so?
    c) How do you generally feel about the tasks associated with immunizations?

3) How do you feel about how immunization records are reported?
    a) How do you feel about overall system for immunizations?
    b) What about management of immunizations?

4) Now, I would like to get a better sense of your work around immunizing children under the age of two. Can you walk through a typical day of conducting immunizations for children under the age of two? [Probe on what activities they do to prepare for immunizations, conduct immunizations, document immunizations, educate around immunization]
    For each step/activity?
    a) Why do you take that particular step?
    b) Who is typically involved in that step?
    c) What you feel when going through that step?
    d) What factors make that step easier/harder?
    e) Can you guide me through the steps that you took today when administering a vaccination?
    f) Are there any cases where you would not administer a vaccination to a child? Why or why not?

5) Are there other, non-typical ways that you provide immunizations?
    a) Can you tell me about those?

For peer review only

6) Outside of conducting immunizations, what else do you do?
    a) Can you tell me about how under-two vaccinations fit into this workload?

7) What is challenging about administering under-two vaccinations?
    a) Did you encounter any of those challenges over the past month? If so, which ones? How did that make your job more challenging?

8) What factors make it easier for you to administer vaccinations?
    a) Did you encounter any of those factors over the past month? If so, which ones? How did that make your job easier?

9) What are your personal beliefs around vaccinations?

10) Can you describe the conversations you usually have with caregivers while administering vaccines?
    a) What about how you see your role in providing advice or convincing caregivers to vaccinate their children under the age of two?

11) What do you view as the primary drivers of under-two immunization drop-outs?
    a) Can you provide an example?

For peer review only

Appendix D: Photovoice Instructional Materials

A CÂMERA

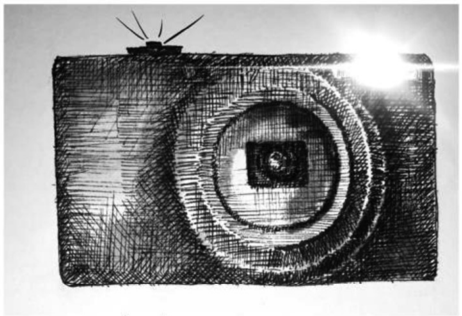

1. Frente da câmera

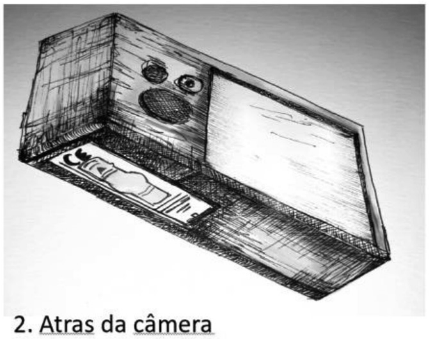

2. Atras da câmera

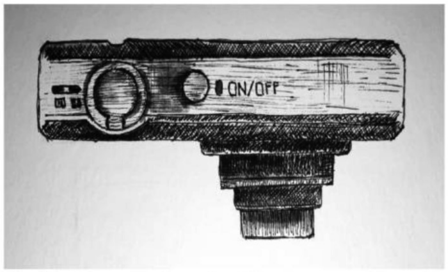

3. Parte superior da câmera

SEGURAR A CÂMERA PARA TIRAR PHOTOS

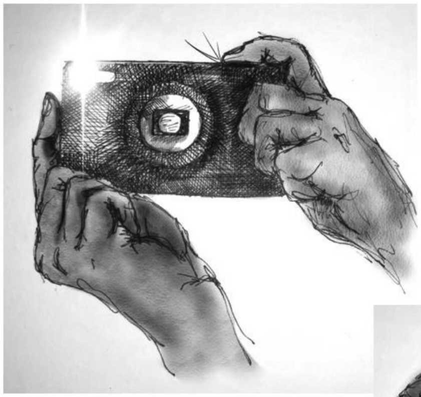

1. Uma Maneira

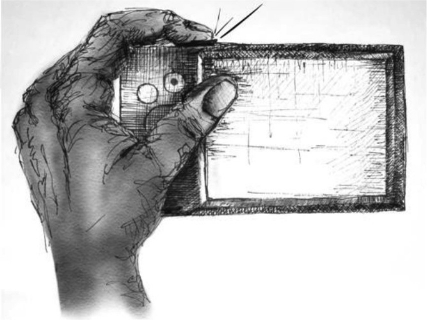

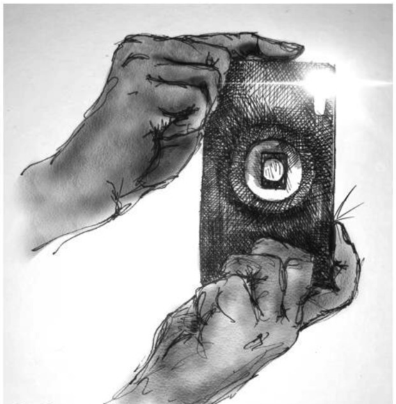

2. Outra Maneira

BATERIA DA CÂMERA

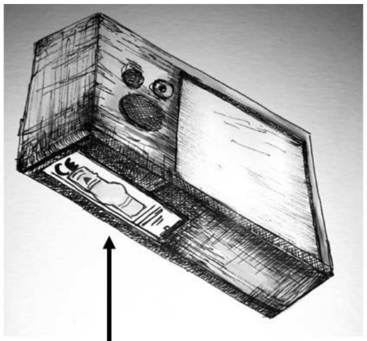

1. Localização da bateria

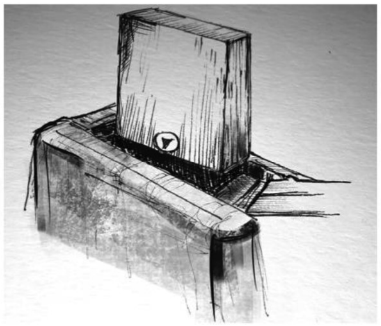

2. Abra o local da bateria

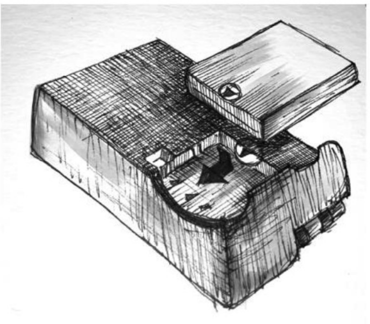

3. Carrega a bateria no carregador

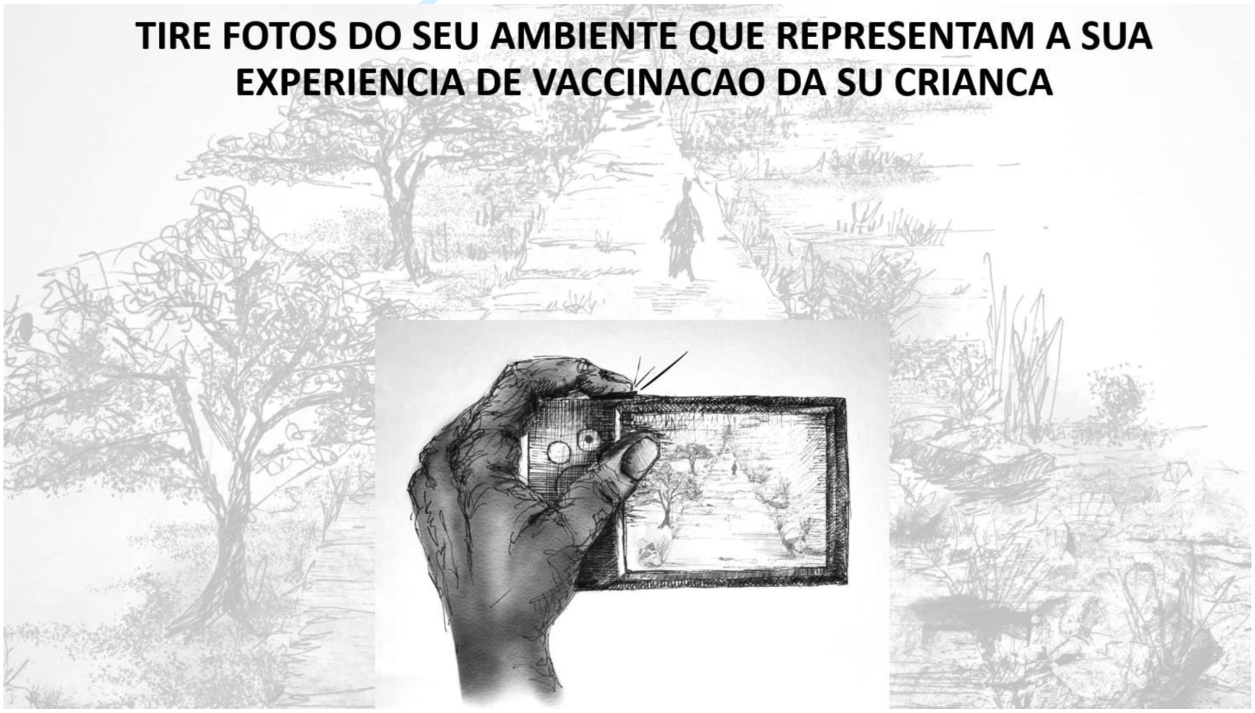

Standards for Reporting Qualitative Research (SRQR)*

http://www.equator-network.org/reporting-guidelines/srqr/

Title and abstract

	Page/line no(s).
**Title** - Concise description of the nature and topic of the study Identifying the study as qualitative or indicating the approach (e.g., ethnography, grounded theory) or data collection methods (e.g., interview, focus group) is recommended	1/1-2
**Abstract** - Summary of key elements of the study using the abstract format of the intended publication; typically includes background, purpose, methods, results, and conclusions	2/1-28

Introduction

**Problem formulation** - Description and significance of the problem/phenomenon studied; review of relevant theory and empirical work; problem statement	3/2-30
**Purpose or research question** - Purpose of the study and specific objectives or questions	3/30-33

Methods

**Qualitative approach and research paradigm** - Qualitative approach (e.g., ethnography, grounded theory, case study, phenomenology, narrative research) and guiding theory if appropriate; identifying the research paradigm (e.g., postpositivist, constructivist/ interpretivist) is also recommended; rationale**	3/37-40
**Researcher characteristics and reflexivity** - Researchers' characteristics that may influence the research, including personal attributes, qualifications/experience, relationship with participants, assumptions, and/or presuppositions; potential or actual interaction between researchers' characteristics and the research questions, approach, methods, results, and/or transferability	4/1-2<br>5/16-22
**Context** - Setting/site and salient contextual factors; rationale**	4/8-14
**Sampling strategy** - How and why research participants, documents, or events were selected; criteria for deciding when no further sampling was necessary (e.g., sampling saturation); rationale**	4/15-24<br>5/6-14
**Ethical issues pertaining to human subjects** - Documentation of approval by an appropriate ethics review board and participant consent, or explanation for lack thereof; other confidentiality and data security issues	5/25-28<br>6/25-28<br>7/1-3
**Data collection methods** - Types of data collected; details of data collection procedures including (as appropriate) start and stop dates of data collection and analysis, iterative process, triangulation of sources/methods, and modification of procedures in response to evolving study findings; rationale**	5/23-36<br>6/1-18

For peer review only

**Data collection instruments and technologies** - Description of instruments (e.g., interview guides, questionnaires) and devices (e.g., audio recorders) used for data collection; if/how the instrument(s) changed over the course of the study	5/23-37<br>6/1-18
**Units of study** - Number and relevant characteristics of participants, documents, or events included in the study; level of participation (could be reported in results)	7/6-16
**Data processing** - Methods for processing data prior to and during analysis, including transcription, data entry, data management and security, verification of data integrity, data coding, and anonymization/de-identification of excerpts	6/4-6<br>6/17-18<br>6/20-25
**Data analysis** - Process by which inferences, themes, etc., were identified and developed, including the researchers involved in data analysis; usually references a specific paradigm or approach; rationale**	6/20-33
**Techniques to enhance trustworthiness** - Techniques to enhance trustworthiness and credibility of data analysis (e.g., member checking, audit trail, triangulation); rationale**	6/22-24<br>6/29-30

Results/findings

**Synthesis and interpretation** - Main findings (e.g., interpretations, inferences, and themes); might include development of a theory or model, or integration with prior research or theory	8/2-13<br>Pages 10-13
**Links to empirical data** - Evidence (e.g., quotes, field notes, text excerpts, photographs) to substantiate analytic findings	Pages 8-9

Discussion

**Integration with prior work, implications, transferability, and contribution(s) to the field -** Short summary of main findings; explanation of how findings and conclusions connect to, support, elaborate on, or challenge conclusions of earlier scholarship; discussion of scope of application/generalizability; identification of unique contribution(s) to scholarship in a discipline or field	13/36-38<br>14/1-28
**Limitations** - Trustworthiness and limitations of findings	15/10-20

Other

**Conflicts of interest** - Potential sources of influence or perceived influence on study conduct and conclusions; how these were managed	15/36
**Funding** - Sources of funding and other support; role of funders in data collection, interpretation, and reporting	16/2-3

*The authors created the SRQR by searching the literature to identify guidelines, reporting standards, and critical appraisal criteria for qualitative research; reviewing the reference lists of retrieved sources; and contacting experts to gain feedback. The SRQR aims to improve the transparency of all aspects of qualitative research by providing clear standards for reporting qualitative research.

For peer review only

For peer review only - http://bmjopen.bmj.com/site/about/guidelines.xhtml

**The rationale should briefly discuss the justification for choosing that theory, approach, method, or technique rather than other options available, the assumptions and limitations implicit in those choices, and how those choices influence study conclusions and transferability. As appropriate, the rationale for several items might be discussed together.

Reference:
O'Brien BC, Harris IB, Beckman TJ, Reed DA, Cook DA. **Standards for reporting qualitative research: a synthesis of recommendations.** *Academic Medicine*, Vol. 89, No. 9 / Sept 2014 DOI: 10.1097/ACM.0000000000000388

For peer review only

For peer review only - http://bmjopen.bmj.com/site/about/guidelines.xhtml

BMJ Open

Determinants of immunization dropout among children under the age of two in Zambézia Province, Mozambique: A community-based participatory research study using Photovoice

Journal:	*BMJ Open*
Manuscript ID	bmjopen-2021-057245.R1
Article Type:	Original research
Date Submitted by the Author:	28-Dec-2021
Complete List of Authors:	Powelson, Jocelyn; VillageReach<br>Magadzire, Bvudzai; VillageReach<br>Draiva, Abel; VillageReach<br>Denno, Donna; University of Washington, Department of Pediatrics<br>Ibraimo, Abdul; VillageReach<br>Benate, Bonifácia; Independent<br>Jahar, Lídia; Independent<br>Marrune, Zélia; Independent<br>Chilundo, Baltazar; VillageReach<br>Chinai, Jalilo; Zambézia Provincial Directorate of Health<br>Emerson, Michelle; VillageReach<br>Beima-Sofie, Kristin; University of Washington School of Public Health, Department of Global Health<br>Lawrence, Emily; VillageReach
**Primary Subject Heading**:	Public health
Secondary Subject Heading:	Global health
Keywords:	PUBLIC HEALTH, QUALITATIVE RESEARCH, International health services < HEALTH SERVICES ADMINISTRATION & MANAGEMENT, Paediatric infectious disease & immunisation < PAEDIATRICS

SCHOLARONE™
Manuscripts

BMJ

I, the Submitting Author has the right to grant and does grant on behalf of all authors of the Work (as defined in the below author licence), an exclusive licence and/or a non-exclusive licence for contributions from authors who are: i) UK Crown employees; ii) where BMJ has agreed a CC-BY licence shall apply, and/or iii) in accordance with the terms applicable for US Federal Government officers or employees acting as part of their official duties; on a worldwide, perpetual, irrevocable, royalty-free basis to BMJ Publishing Group Ltd ("BMJ") its licensees and where the relevant Journal is co-owned by BMJ to the co-owners of the Journal, to publish the Work in this journal and any other BMJ products and to exploit all rights, as set out in our licence.

The Submitting Author accepts and understands that any supply made under these terms is made by BMJ to the Submitting Author unless you are acting as an employee on behalf of your employer or a postgraduate student of an affiliated institution which is paying any applicable article publishing charge ("APC") for Open Access articles. Where the Submitting Author wishes to make the Work available on an Open Access basis (and intends to pay the relevant APC), the terms of reuse of such Open Access shall be governed by a Creative Commons licence – details of these licences and which Creative Commons licence will apply to this Work are set out in our licence referred to above.

Other than as permitted in any relevant BMJ Author's Self Archiving Policies, I confirm this Work has not been accepted for publication elsewhere, is not being considered for publication elsewhere and does not duplicate material already published. I confirm all authors consent to publication of this Work and authorise the granting of this licence.

For peer review only

Determinants of immunization dropout among children under the age of two in Zambézia Province, Mozambique: A community-based participatory research study using Photovoice

Authors

Jocelyn Powelson, VillageReach, Seattle, USA

Bvudzai Magadzire, VillageReach, Cape Town, South Africa

Abel Draiva, VillageReach, Quelimane, Mozambique

Donna M. Denno, Department of Pediatrics, University of Washington, Seattle, USA

Abdul Ibraimo, VillageReach, Maputo, Mozambique

Bonifácia Beleza Lucas Benate, Independent, Namarroi, Mozambique

Lídia Carlos Jahar, Independent, Gilé, Mozambique

Zélia Marrune, Independent, Gilé, Mozambique

Baltazar Chilundo, VillageReach, Maputo, Mozambique

Jalilo Ernesto Chinai, Zambézia Provincial Directorate of Health, Quelimane, Mozambique

Michelle Emerson, VillageReach, Seattle, USA

Kristin Beima-Sofie, Department of Global Health, University of Washington, Seattle, USA

Emily Lawrence, VillageReach, Seattle, USA

Corresponding Author

Bvudzai Magadzire

210 S Hudson St, Suite 307, Seattle, WA 98134

bvudzai.magadzire@villagereach.org

Word Count

5,746

For peer review only

ABSTRACT

Objective: Immunizations are highly impactful, cost-effective public health interventions. However, substantial gaps in complete vaccination coverage persist. We aimed to describe caregivers' immunization experiences and identify determinants of vaccine dropout.

Design: We used a community-based participatory research approach employing Photovoice, SMS exchanges, and in-depth interviews. A team-based approach was used for thematic analysis. The Increasing Vaccination Model guided the analysis and identification of vaccination facilitators and barriers.

Setting: This study was conducted in Zambézia Province, Mozambique, in Namarroi and Gilé Districts, where roughly 19% of children under 2 start but do not complete the recommended vaccination schedule.

Participants: Participants were identified through health facility vaccination records and included caregivers of children aged 25-34 months who were fully-vaccinated (n=10) and partially-vaccinated (n=22). We also collected data from 12 health workers responsible for delivering immunizations at the selected health facilities.

Results: Four main patterns of barriers leading to dropout emerged: 1) social norms and limited family support place the immunization burden on mothers; 2) perceived poor quality of health services reduces caregivers' trust in vaccination services; 3) concern about side effects causes vaccine hesitancy; and 4) caregivers hesitate to seek and advocate for vaccination due to power imbalances with health workers. COVID-19 created additional barriers related to social distancing, mask requirements, supply chain challenges, and disrupted outreach services. For most caregivers, dropout becomes increasingly likely with compounding barriers. Caregivers of fully-vaccinated children noted facilitators, including accompaniment to health facilities or assistance caring for other children, which enabled them to complete vaccination.

Conclusions: Overcoming immunization barriers requires strengthening health systems, including improving logistics to avert vaccine stockouts and building health worker capacity, including empathic communication with caregivers. Consistent and reliable immunization outreach services could address access challenges and improve immunization uptake, particularly in distant communities.

For peer review only

Strengths and limitations of this study

- Photovoice methods allowed participants to visually share their stories and perspectives, generating rich, detailed, and nuanced information about the barriers that they experience in fully immunizing their children.
- The community-based participatory research approach allowed community members to engage throughout the research stages, ensuring that the findings and resulting recommendations are community-centered.
- Due to the contextual nature of vaccination systems and vaccination barriers, the results may have limited generalizability beyond similar settings and populations.
- The COVID-19 pandemic prevented us from convening any focus groups and may have affected the quality of our participatory analysis activities, which had to be conducted virtually rather than in-person.

INTRODUCTION

Childhood immunizations are highly impactful and cost-effective public health interventions, estimated to annually save 2-3 million lives worldwide.[1] In 1974, the World Health Organization (WHO) established the Expanded Program on Immunization (EPI) to guide the development and implementation of vaccination programs globally. Since then, coverage of the initial vaccines (protecting against diphtheria, tetanus, pertussis, measles, poliomyelitis, and tuberculosis) has increased from fewer than 5% of children in low- and middle-income countries (LMICs) to more than 85%.[2,3] However, significant gaps in vaccination coverage remain. In 2019, an estimated 14 million children were unimmunized, and 5.7 million were under-immunized,[4] predominantly from lower socioeconomic and rural populations in LMICs.[5] The COVID-19 pandemic has reversed recent progress, with an estimated 23 million children not completing basic vaccinations in 2020.[6]

The Mozambique EPI, launched in 1979, provides immunization services free of charge. Public sector health workers deliver EPI vaccines at fixed health posts, through routine mobile outreach activities, and sometimes during campaigns for specific vaccines.[7] Since the early 2000's, Mozambique has implemented several strategies, including the WHO/United Nations Children's Fund (UNICEF) Reaching Every District/Reaching Every Community (RED/REC) strategy, to improve vaccination outreach in remote communities.[7] According to the Mozambique EPI Manual, mobile vaccination brigades should provide services quarterly to communities residing further than eight kilometers from a health facility.[8] These outreach events are important for facilitating access to vaccines, as roughly 65% of the country's population lives in rural areas.[9] However, country-wide implementation of immunization outreach strategies has been impeded by insufficient funding and limited material and human resources, resulting in coverage gaps.[7]

Routine vaccination coverage in Mozambique increased dramatically from 47% in 1997 to 63% in 2003, but progress has since slowed; according to the 2015 Survey of Indicators on Immunization, Malaria, and HIV/AIDS in Mozambique, only 66% of children were fully vaccinated,[10] and in 2019, UNCIEF and WHO estimated the third dose of the Diptheria-Tentanus-Pertussis (DPT 3) vaccine (a common proxy for complete immunization coverage) at 88%.[1] Within Mozambique, Zambézia Province has the lowest vaccination coverage; in 2015, 50% of children under 2 had received the first doses of recommended immunizations, and 38% had started but dropped out from the vaccination schedule.[10] Low coverage of the first set of recommended vaccines indicates poor access to services or lack of vaccination acceptance, while high dropout rates suggest health system failures to successfully deliver repeated doses.[11] Low dropout rates are critical to preventing morbidity and mortality from vaccine-preventable diseases.[12] In the context of Zambézia Province, this is particularly critical as there have recently been reported outbreaks of vaccine-preventable diseases such as cholera, polio, and measles.[13] In Mozambique, there is a dearth of knowledge on drivers of routine immunization dropouts specifically, as opposed to children who do not receive *any* of the routine immunizations. Determinants of immunization dropout have been shown to be highly contextual, including combinations of individual, inter-personal, and health systems factors.[14] We conducted interviews with caregivers of young children and health workers, using a community-based participatory research (CBPR) approach, to identify key influences on under-2 immunization dropout in two districts in Zambézia Province, Mozambique.

METHODS

For peer review only - http://bmjopen.bmj.com/site/about/guidelines.xhtml

Study Design

We conducted a qualitative study using CBPR and Photovoice methods. CBPR engages community representatives throughout the research process, reducing power imbalances between researchers and participants, creating an environment in which participants feel comfortable discussing sensitive topics, and facilitating the co-creation of contextually-sensitive and community-centered knowledge.[15] Four Mozambican caregivers (referred to as Caregiver Researchers) were recruited from the local communities, were trained in research ethics and qualitative methods, led data collection, and assisted with data analysis.

The Increasing Vaccination Model, developed by the WHO Behavioral and Social Drivers of Vaccination (BeSD) working group,[16] guided data collection and analysis. We adapted this model to incorporate elements of the UNICEF Caregiver Journey Model, dividing the "practical issues" category into three sub-categories to capture the vaccination timespan (pre-, during-, and post-service) (Appendix A).

Study Setting, Population, and Recruitment

The study was conducted between February 2020 and March 2021 in Gilé and Namarroi Districts, Zambézia Province. Zambézia, the second-most populated province in Mozambique, has many hard-to-reach communities with some of the lowest health outcomes; a child born in Zambézia is twice as likely to die before age five than a child born in the capital city of Maputo.[7,17] Of the province's 16 districts, Namarroi and Gilé have the highest under-immunization rates, both at roughly 19%.[18] Gilé District has 11 fixed health facilities, serving a population of roughly 205,000.[18] Namarroi District has 10 fixed health facilities, serving a population of approximately 155,000.[18]

The study population included caregivers of fully- or partially-vaccinated children who lived in catchment areas of the selected health facilities, and health workers who administer immunizations at those facilities. 'Caregivers,' defined as parents or other guardians who take primary healthcare-seeking responsibility for the child, were eligible to participate if their child was 25-34 months old, an age by which the child should have received all routine childhood immunizations according to the Mozambique immunization schedule (Appendix B). Caregivers were considered to be in the fully-vaccinated group (FV caregivers) if their child had received all 15 recommended immunizations, or the partially-vaccinated group (PV caregivers) if their child had received at least one but not all recommended immunizations. FV caregivers were included in the study to allow for comparison of experiences and barriers between PV and FV caregivers. Health workers were eligible if they worked at the selected health facilities and if they were responsible for administering vaccinations to children under 2. Health workers were included in the study to provide additional perspective and nuance on the barriers that caregivers described.

We purposively selected eight health facilities (four per district) as recruitment sites to achieve a mixture of rural and peri-urban environments. Eligible caregivers were identified based on the facilities' immunization records. Potential caregiver participants were excluded if they resided outside the health facility catchment area or their vaccination status did not meet definitions for partial or full vaccination. Caregiver Researchers then used a convenience sampling approach to visit caregivers' homes until they reached targeted enrollment numbers of approximately 20 PV caregivers and 10 FV caregivers. During recruitment, we verified vaccination status via home-based vaccination cards, which were considered more accurate when there were discrepancies with health facility records. Sixteen caregivers from each district were enrolled (Figure 1), whereupon data saturation was reached. The study team purposively

recruited and enrolled six health workers from each district, including at least one from each of the selected health facilities. No health workers declined to participate.

Data Collection

Caregiver Researchers were recruited via an open job posting advertised in Namarroi and Gilé Districts. Applicants were required to have a 24-36 month-old child, and women were prioritized, as they were more representative of the study population. Three women from each district were selected based on these requirements and on their level of education, fluency in local languages, and prior research experience. They participated in a five-day in-person training on qualitative research methods, and contracts were extended to the two women from each district with the best performance during the training.

Guides for caregiver and health care worker semi-structured interviews were developed in English based on the Increasing Vaccination Model and translated into Portuguese (Appendices C and D). The guides were piloted with the Caregiver Researchers and one health worker, resulting in minor modifications to improve question clarity. Caregiver Researchers provided participants with study information. Basic demographic information was collected from all participants prior to each interview. Illiterate participants provided thumbprints instead of signatures, otherwise written consent was obtained, including for use of photographs of them and their children.

Photovoice and semi-structured interviews were used to document caregivers' experiences with the under-2 immunization process and to identify immunization completion barriers and facilitators. Photovoice, a method through which people can share their stories using photographs that visually represent their experiences and perspectives, can empower participants to craft rich stories, generating data that might not be captured through standard interview techniques.[19,20] Caregiver Researchers provided cameras to caregiver participants, gave detailed instructions on their use (Appendix E), and asked caregivers to take photos related to their experiences immunizing their child. Caregiver Researchers returned after two to six days and conducted audio-recorded, in-depth interviews. Each caregiver selected the five photos that they felt represented the most meaningful depictions relating to their immunization experience and began the interview by explaining the meaning and importance of each photo. Photo explanations were followed by a semi-structured set of questions for all caregivers to gather additional data. Initial interviews and transcripts were reviewed by EL and JP, who provided feedback and additional training to improve quality of subsequent interviews. Interviews were conducted in the local language of Elomué and lasted an average of 38 minutes. Following each interview, Caregiver Researchers completed written interview debriefs, noting key insights. Audio-recorded caregiver interviews were transcribed and translated from Elomué to Portuguese by Caregiver Researchers, audited for quality by study team members, and then translated into English by an external translator and ME.

SMS exchanges and semi-structured interviews were used to document health workers' perceptions of caregiver immunization experiences, their beliefs about causes of dropout, and their experiences administering vaccines to children under 2. SMS exchanges took place during January and February 2021. Each health worker was provided a phone with the Telegram application set up for group chats with the Caregiver Researchers and study team members. Over a three-week period, health workers were asked to send photos and messages whenever they had an experience or observation related to under-2 immunization. Caregiver Researchers replied with follow-up probes to collect additional information. If more than two days elapsed without any messages exchanged, the Caregiver Researchers sent follow-up

For peer review only

reminders. On average, each health worker sent 33 messages (range 11-51 messages). In February 2021, Caregiver Researchers conducted audio-recorded interviews by phone with health workers in Portuguese, lasting an average of 70 minutes. Health worker interview audio recordings and SMS exchanges were transcribed and translated from Portuguese to English by an external translator.

Data Analysis

A team-based approach was used to perform a thematic analysis.[21] An initial codebook was developed deductively based on the Increasing Vaccination Model. Additional codes were added inductively following review of the first six transcripts from caregiver interviews, and the codebook was reviewed and updated by the Caregiver Researchers prior to coding. JP and EL individually coded the first three caregiver interview transcripts in ATLAS.ti Version 9, and BM reviewed the coded transcripts for intercoder agreement. JP and EL then divided and independently coded the remaining caregiver interview transcripts and summarized the main findings by vaccination status. Upon review of the first three transcripts from health worker SMS exchanges and interviews, additional codes were added to the codebook to capture the unique perspectives and experiences described by health workers. Health worker SMS exchanges and interview transcripts were coded by JP and EL and findings were summarized by JP, EL, BB, AI, LJ, and ZM.

Caregiver Researchers participated in a virtual two-week participatory analysis process facilitated by Portuguese-speaking study team members (AI, ME), in which they reviewed code summaries and representative quotations, identified themes and key barriers to vaccination, and drew comparisons between PV and FV caregivers and between caregiver and health worker perspectives. An online data collaboration platform (Miro, 2021) was used to facilitate collaboration and establish consensus on themes.

The Photovoice photographs provided additional insight on what caregivers believed were the most significant aspects of their vaccination journeys. Each photo selected by the caregivers (including 110 photos from PV caregivers and 50 photos from FV caregivers) was reviewed and classified according to the subject of the photo, and the most common photo subjects of PV and FV caregivers' photos were compared. JP and EL reviewed the themes generated in the participatory analysis workshop and the photos to identify dropout patterns related to the Increasing Vaccination Model.

Patient and Public Involvement

The Caregiver Researchers, as representatives of the study population, participated in data collection and analysis. Study results were disseminated to most participants during follow-up workshops in April and May 2021. Participants will be involved in dissemination of results back to their communities and to local health authorities.

Ethics Statement

The Mozambique National Bioethics Committee for Health approved the study procedures (Ref:259/CNBS/20) and the University of Washington Institutional Review Board exempted the study from review (STUDY00011999).

RESULTS

Participants

For peer review only

Thirty-two caregivers participated in the study, and all were the mothers of the children. The majority lived in catchment areas of rural health facilities. On average, PV caregivers were older and less educated than FV caregivers (Table 1). Partially-vaccinated children were missing an average of 5.1 vaccines (range 1-14 missing vaccines), with the most commonly missed vaccines being Inactivated Polio and Measles-Rubella (Tables 1 and 2).

Table 1: Characteristics of caregiver participants and their children

	Partially-vaccinated (n=22) N (%) or median (IQR)	Fully-vaccinated (n=10) N (%) or median (IQR)
District		
Namarroi	11 (50%)	5 (50%)
Gilé	11 (50%)	5 (50%)
Health facility geography		
Rural	16 (73%)	7 (70%)
Peri-urban	6 (27%)	3 (30%)
Caregiver age (years)	26 (22-30)	21 (20-21.8)
Caregiver education		
Some primary	11 (50%)	3 (30%)
Completed primary	11 (50%)	7 (70%)
Child age (months)	30 (30-32)	31.5 (29.3-32)
Child sex		
Female	9 (41%)	6 (60%)
Number of missing vaccines (of 15 possible)	3.5 (2-8.8)	0

Table 2: Vaccination completion of the partially-vaccinated children of caregiver participants

**Vaccine**	**Dose**	**Number (%) of partially-vaccinated children who received the dose (n=22)***
Tuberculosis	BCG	22 (100%)
Oral Polio	OPV 0	15 (68.2%)
	OPV 1	19 (86.36%)
	OPV 2	15 (68.2%)
	OPV 3	12 (54.6%)
Pentavalent: Diphtheria, Pertussis, Tetanus, Hepatitis B, Haemophilus influenzae type B	DPT-HepB-Hib 1	20 (90.1%)
	DPT-HepB-Hib 2	15 (68.1%)
	DPT-HepB-Hib 3	13 (59.1%)
Pneumococcal conjugate	PCV 1	21 (95.45%)
	PCV 2	17 (77.3%)
	PCV 3	12 (54.6%)
Rotavirus	RV 1	17 (77.3%)
	RV 2	12 (54.6%)
Inactivated Polio	IPV	7 (31.8%)
Measles, Rubella	MR 1	3 (13.6%)
	MR 2**	3 (13.6%)

*Note: Vaccination status is according to vaccination cards, or health facility records when no cards were available. Twins were counted as a single child.

For peer review only

**Note: The second dose of MR was added to the Mozambique EPI schedule in November 2017. Due to the timing of the study, completion of MR 2 was not considered when determining vaccination status.

Twelve health workers participated in the study (Table 3). Most were male and had completed secondary education. Health workers had worked at the selected health facilities for an average of 4.7 years. All had children of their own, including five with children under 2.

Table 3: Characteristics of health worker participants

	N (%) or median (IQR)
District	
Namarroi	6 (50%)
Gilé	6 (50%)
Education Level	
Completed secondary	11 (92%)
Completed primary	1 (8%)
Work Experience	
Total years of work experience	7 (4.5-9.3)
Years at selected health facility	4 (2-5)
Job title	
Preventive Medical Technician	6 (50%)
General Nurse	4 (33%)
Maternal and Child Health Nurse	1 (8%)
Nutrition Technician	1 (8%)
Immunization trainings	
Attended immunization-specific training within past 5 years	5 (42%)
Attended immunization-specific training 5 or more years ago	2 (17%)
No formal trainings but met with colleagues to discuss introduction of new vaccines	2 (17%)
Never participated in vaccination trainings	3 (25%)
Sex	
Male	9 (75%)

For peer review only

Photovoice Findings

Photovoice outputs depicted the people, places, and things that caregivers felt were most important in their vaccination journeys. There were several notable differences between the most common subjects of PV and FV caregivers' photos. Sixty-four percent of PV caregivers, versus only 30% of FV caregivers, selected at least one photo depicting them caring for their child who was experiencing side-effects after the vaccination, and many voiced concerns about these side-effects. Conversely, 60% of FV caregivers selected at least one photo of their own child or other children in the community who were healthy and growing well due to vaccines, a subject that only 27% of PV caregivers photographed. Roughly half of caregivers in both groups took photos of family and friends, but they described those photos differently; FV caregivers were much more likely to describe active support (e.g. family accompanying them to the health facility), while PV caregivers typically described only passive encouragement from family or talked

about times when family members did not provide help with the vaccination process. Roughly half of caregivers in both groups also took photos of bathing the child before vaccination and of walking to the health facility while carrying the child.

Key Influences on Vaccine Dropout

Vaccination barriers arose in all categories of the Increasing Vaccination Model. In general, PV caregivers described abandoning vaccination after encountering multiple cross-domain barriers, rather than dropout being caused by a single barrier.

> *"…my father's illness until death, in addition to my illness, money and not even someone to help me, these are the events that happened during the first two years of my son's life." – PV Caregiver from Gilé*

Our analysis of caregiver experiences and photos, coupled with insight from health workers, revealed four cross-domain patterns of barriers and facilitators to vaccination completion. These patterns related to, 1) social norms and limited family support; 2) perceived poor quality of health services; 3) concern about side effects; and 4) power imbalances between caregivers and health workers (Table 4). COVID-19 created additional barriers to vaccination completion. Unless otherwise indicated, the findings described below relate to both PV and FV caregivers.

Table 4: Cross-domain influences on vaccine dropout

**Patterns of barriers and facilitators to vaccination completion**	**Increasing Vaccination Model categories**	**Specific barriers and facilitators**	**Illustrative quotation**
Social norms and limited family support place the burden of vaccination on mothers, compounding the challenges of accessing vaccination services.	• Social processes<br>• Practical issues: Preparation	• Long distance to health facility<br>• Physically challenging journey to vaccinate<br>• Caregiver illness or injury<br>• Family support<br>• Women's role in society	*"I came back from the hospital and sat here on the mat… My leg was in a lot of pain again on my way to the hospital and I ended up not vaccinating [my child]. I'm not going there anymore…. When I ask my husband to accompany our child to the hospital, he says that he is not able to take the child on his back..." – PV Caregiver from Namarroi*
Perceived poor quality of health services reduces caregivers' trust in the health system.	• Motivation<br>• Practical issues: Preparation<br>• Practical issues: Point of care	• Caregiver dissatisfaction with health services<br>• Vaccine stockouts<br>• Lack of health care workers	*"Sometimes when we arrived at the hospital, they claimed that there were no vaccines, so the child was just weighed…. When that happened, I'd regret the distance I had to travel…. The following month I would not return with fear of spending money and not finding the vaccine." – PV Caregiver from Gilé*

Concern about side effects, exacerbated by "accumulation" of vaccines, leads to hesitancy.	• What people think and feel<br>• Practical issues: Point of care<br>• Practical issues: After care	• Fear of vaccine "accumulation"<br>• Lack of information about vaccine, side effects and schedule<br>• Perception that side effects are normal	*"The child caught fevers [after vaccination… Sometimes she had two or three days with fevers and swollen legs. She got more fevers in the months that I had multiple vaccines at the same time. As I was not informed about the side effects of getting different vaccines at the same time, the child would get home with many fevers and worry me." – PV Caregiver from Gilé*
Power dynamics at the health facility make caregivers hesitant to seek or advocate for vaccination.	• Social processes<br>• Motivation<br>• Practical issues: Preparation<br>• Practical issues: Point of care	• Out-of-schedule vaccinations<br>• Hygiene<br>• Vaccination cards<br>• Vaccination session hours	*"Whenever I take my child to the hospital to vaccinate, I first bathe to avoid rejection and other illnesses that can be caught due to poor hygiene. Since when you take the child without bathing, it will not be vaccinated, it is refused.… When taking the child without bathing, I am afraid that other mothers will laugh and consider me as a dirty person." – PV Caregiver from Gilé*

Social Norms and Limited Family Support Place the Burden of Vaccination on Mothers, Compounding the Challenges of Accessing Vaccination Services

For many caregivers, traveling to a health facility required significant investments of time, effort, and money. Some caregivers described selling crops or buying less food to save money for transport to the health facility. When they had no money for transportation, caregivers described having to walk for several hours while carrying their children, often through challenging and dangerous terrain and inclement weather, even spending the night because the trip was too long to complete in a single day.

> *"The hospital is very distant. On the days we have to go on foot, we come home with our feet swollen from so much walking. There are times when you cannot go home because it is late, and you are forced to sleep there, even with nowhere to stay." – PV Caregiver from Gilé*

PV caregivers noted that given the long distances required to travel to receive vaccinations, lack of support from other family members was a major barrier to completing the vaccination schedule. Limited family support was in part due to community beliefs that vaccination responsibility resides with mothers and that fathers should not be involved in the process. This belief resulted in some mothers abandoning the process when they were unable to travel to the facility due to illness or injury, and their husbands and other family members did not help bring the children to the health facility. Several other mothers reported dropping out due to lack of support with managing other conflicting obligations, such as caring for other children.

For peer review only

> *"[My aunt] had a toothache and couldn't stay with [my other children]. When she told me this, I was left with no way out; I couldn't go to the hospital and leave my children alone." – PV Caregiver from Gilé*

Social support from family and friends helped FV caregivers to overcome vaccination barriers. FV caregivers described receiving support in a variety of ways including reminders from family members, money for transport to the health facility, accompaniment to the facility by other caregivers or a family member, help with other household responsibilities, and support in taking the child to the facility when the mother is sick.

Both caregivers and health workers noted that reliable, consistent mobile brigades would help to address the practical barriers associated with traveling to health facilities, especially for those mothers who lack support from family members. Several caregivers mentioned that mobile brigades had previously enabled them to complete vaccinations of older children, but that the brigades had become infrequent and unreliable. Health workers described barriers to running mobile brigades, including insufficient fuel and lack of staffing to manage both fixed posts and outreach services.

> *"We need motorcycles and fuel so that we can run mobile brigades in the communities.... There are times when this doesn't happen 100% because the bikes are damaged, and we can't do maintenance.... When we do not have funds for fuel, sometimes the mobile brigades are paralyzed." – HW from Namarroi*

Perceived Poor Quality of Health Services Reduces Caregivers' Trust in the Health System

Many caregivers began the vaccination process highly motivated, and some described having positive experiences at health facilities. However, other caregivers were dissatisfied with their experiences at the service delivery point due to vaccine stockouts, long wait times, and inconsistent health facility hours. PV caregivers also described being frustrated by interactions with disengaged health workers. After expending significant effort to reach the health facility, some caregivers arrived to find that health workers were late or were busy talking on their phones, causing them to lose faith in the health system and lose motivation to vaccinate.

> *"I arrived at the hospital at a good time, before the activities started. I was waiting for the nurses until the time they arrived.... I was very indignant, since the nurses attended a person and interrupted to answer telephone calls that lasted many hours without consideration for mothers who live very far from the hospital." – PV Caregiver from Gilé*

These dissatisfying experiences extended to general health services too; several PV caregivers discussed visits for treatment of malaria or other illnesses in which they received only partial doses of medications, leading them to also question the quality of vaccination services and abandon the vaccination process, rather than continue to invest time, effort, and money.

Health workers described their own challenges in delivering vaccines and providing quality care to every child, including stockouts of vaccines and other supplies and high work burden due to insufficient human resources. Many health workers described vaccine stockouts lasting weeks to months with no guarantee of when doses would be available again. Several health workers raised concerns that aligned with those expressed by caregivers: that caregivers would not return after encountering stockouts due to distances and other difficulties associated with vaccine care seeking.

For peer review only

"The whole province… has been out of stock of [the polio vaccine] for 2 months.… Considering that the vaccination program has a certain calendar, it is always embarrassing that this happens, because the children who should have had vaccinations in that period do not get them." – HW from Gilé

Concern about Side Effects, Exacerbated by "Accumulation" of Vaccines, Leads to Hesitancy

Some caregivers understood that side effects such as swelling at the injection site or fever are common and should not be a reason to stop vaccination. However, many PV caregivers expressed concerns about these reactions, especially when they lasted for several days. This concern was also greater for PV caregivers who lived far away and felt that the long walk home aggravated the side effects. Some PV caregivers worried that "simultaneous" or "accumulated" vaccines – those given to catch up on missed doses -- would exacerbate the fevers and swelling, causing some to drop out of the vaccination process.

"…what made me abandon it were the side effects of the vaccine. I wanted to protect my son by abandoning the service and I didn't know I was harming the child.... On the days that he received the vaccines simultaneously, he would have many fevers. This happened when he spent many months without vaccinating." – PV Caregiver from Gilé

Health workers recognized these concerns. They felt that caregivers might not be aware that reactions are relatively common and are rarely harmful in the long-term, and they noted that better communication with caregivers is important for reducing dropouts caused by fear of side effects.

"[The health worker] needs to sensitize the mother…why the child is getting that vaccine, and what will be the effect of that vaccine. For example, if the child has a fever after that vaccination, you tell the mother not to worry because it is normal..." – HW from Namarroi

Power Dynamics at the Health Facility Make Caregivers Hesitant to Seek and Advocate for Vaccination Services

Several caregivers described negative interactions with health workers. Caregivers feared that health workers would scold them and send them home if they came out of schedule after missed doses, did not bathe their child prior to the visit, or forgot to bring their vaccination card.

"If you have too many absences, when you arrive at the hospital you are insulted for not taking the child for the vaccination, even though you try to explain that it was not of your own will but because of the distance." – PV Caregiver from Gilé

There were strong social norms around bathing the child before vaccination to avoid being rejected or humiliated. PV caregivers' fear of humiliation was exacerbated by the public nature of the health facility, where immunization activities and conversations take place in front of other caregivers due to lack of private rooms.

Caregivers frequently discussed the importance of vaccination cards. Both PV and FV caregivers felt that cards were important for tracking the vaccination schedule and for allowing health workers to easily identify their child's next vaccine. However, PV caregivers also noted how cards could negatively influence treatment and services, describing fears around being verbally abused and rejected by health workers over lost or damaged cards. Caregiver mothers who had given birth outside of a facility saw the cards as

For peer review only

prohibitive of receiving vaccination services, fearing that if they requested a vaccination card, health workers would admonish them for the non-institutional delivery.

> *"[My child] didn't get all the vaccinations because she didn't have a card. When it got torn, I arrived at the hospital to ask for a new card. I was told to get the previous one and I was left with no options." – PV Caregiver from Namarroi*

PV caregivers noted that power dynamics sometimes also resulted in missed vaccination opportunities. A few PV mothers described instances when health workers sent them home without delivering any vaccinations. Even when PV caregivers believed that their children needed to be vaccinated, they were hesitant to approach the health workers to request the vaccine, fearing that they would be yelled at.

> *"Many times when I went to the hospital, they just sent my daughters to weigh and said they had already completed the vaccinations and didn't even check the children's cards. But as a mother, I knew that the last vaccine was missing and I couldn't question it for fear of being humiliated." – PV Caregiver from Gilé*

From the health worker perspective, however, some of these missed opportunities were due to challenges that they faced regarding vaccine delivery and cold chain policies. Some health workers said they do not vaccinate children over the age of two, and one described policies that limit them to opening vaccine freezers just once at the start of the vaccination session and once at the end. If caregivers arrived after the vaccines were put away, the health workers could not retrieve the vaccines. Additional missed opportunities to vaccinate occurred at two health facilities; at one, there was no maternal and child health nurse to manage vaccination of newborns on weekends, and at the other, the maternal and child health nurses had been trained in vaccination but were not administering them to newborns.

> *"All deliveries that take place on the weekends must return on Monday to vaccinate. Sometimes there are missed opportunities because mothers live far away.... [Maternal and child health] nurses have training in vaccination, and it is possible to manage the vaccination of newborns - just apply the BCG vaccine and then dismiss the mothers - but this is not happening." – HW from Gilé*

The data suggest a breakdown in communication between health workers and caregivers; health workers do not always communicate their logistical and resource constraints, and caregivers are afraid to ask about why their children are not receiving vaccines.

> *"For a few months that I went to the hospital they just weighed the children and returned the card to me without saying anything, but I was afraid to ask. I was silent without knowing if it was a lack of vaccines at the hospital or bad luck for me." – PV Caregiver from Gilé*

Impacts of COVID-19 on Vaccination Experiences

Though some caregivers and health workers felt that COVID-19 had not impacted the vaccination process, others described changes to health services and other barriers to vaccination resulting from the pandemic. In some cases, caregivers were hesitant to go to the health facility due to fear of contracting COVID-19 or general discomfort around the new health facility policies. Mask requirements were another reported deterrent, as caregivers who did not have their own masks knew that they would not be served.

> *"In the hospital, without the use of masks, people are not attended, as well as on public transport. Our habits of shaking hands and staying close to our friends is also forbidden. These are things*

For peer review only

that make us distressed and worried without knowing how long it will take before it passes." – PV Caregiver from Gilé

At the health facility level, health workers described shortages of general supplies, such as gloves, and increased vaccine stockouts.

"At times, we lacked some vaccines because…the borders closed…due to COVID-19.… For example, we are currently without BCG and polio vaccines.… There is difficulty in [transporting the vaccines to] the province." – HW from Namarroi

Health workers also mentioned challenges maintaining social distancing because the waiting rooms are too small to accommodate all the caregivers, forcing caregivers and their children to wait outside even in hot sun or rain.

Both caregivers and health workers noted that mobile brigades have been especially infrequent during the COVID-19 pandemic. Health workers described difficulties in organizing mobile brigades due to social distancing requirements as well as lack of fuel and required supplies. Caregivers commented that they were unaware of brigade schedules due to closures of schools and churches, which previously were their main sources of information about brigades. When mobile brigades did happen, health workers reported that far fewer caregivers than usual brought their children due to fears of congregating in groups.

"Now we go to the community, and we find there are not enough children due to the pandemic, and messages are arriving in the communities saying that we cannot concentrate…fifty people together.… If before COVID-19, I could reach 200 children in immunization, today when I go, I can only reach 30 to 50 children." – HW from Namarroi

DISCUSSION

Our study highlighted the immense effort that caregivers put into vaccinating their children and the numerous and difficult barriers they face. Primary barriers identified in our study included: practical issues related to health facility access, unreliable and poorly perceived service quality, negative interactions with health workers, caregiver misunderstandings about vaccine side effects, and unsupportive household dynamics. Our findings are consistent with many of the dropout determinants that have previously been identified in systematic reviews that included LMIC settings, including: access, health worker availability, missed opportunities, service reliability, family and gender dynamics, childcare challenges for siblings, lack of motivation, fear of side-effects, mistrust of the health system, and health staff attitudes and behavior.[14,22–25] Other studies in sub-Saharan Africa have identified specific vaccination barriers very similar to ours, including poor interactions with health workers, such as being verbally abused if the child did not look presentable, as well as incurring expenses to reach a facility only to find vaccine stockouts.[23] Our findings also align with results from cross-sectional studies in Mozambique, which found that dropout was related to lack of access to vaccination facilities, concern about receiving multiple vaccines at once, household decision-making processes, and children born at home or outside of Mozambique.[23,26,27] Additional barriers that have been previously identified in Mozambique, but which did not arise explicitly in this study, were maternal education and poverty, though we did see that illiteracy and cost were barriers. This study also did not find evidence of

For peer review only

vaccination completion relating birth order or number, determinants that have been identified in other African countries.[28]

Our study generated several new findings related to caregivers' interactions with health workers and their perceptions of service quality, caregivers' hesitancy related to vaccine "accumulation," the importance of family support, and the shortcomings of the existing outreach services. Fear of humiliation or reprimand contributed to caregivers' perceptions of poor service and discouraged them from continuing with vaccination once their child had fallen off schedule. The perceived poor quality of *general* health services influenced some caregivers' decisions to stop seeking vaccination services. Communication breakdowns may have made caregivers unaware of health workers' constraints, exacerbating their perceptions of poor service quality. Many caregivers in our study were more fearful of side effects when they believed that their child was receiving too many vaccines simultaneously and especially in the context of catch-up administration. Our findings showed that family support of mothers is a strong enabler to overcoming many vaccination barriers, but the dominant social norms exempt husbands and other family members from assisting with the vaccination process. Finally, the data showed that currently, outreach services are not meeting the needs of caregivers in distant communities; mobile vaccination brigades have been infrequent, especially due to COVID-19, and caregivers do not always receive advance notice of them. Overall, vaccine dropout was related to compounding barriers that eventually tip the scales in favor of abandoning the vaccination cycle. This study has generated a more comprehensive understanding of how those barriers interact together.

Implications on Policy and Practice

Health systems strengthening is receiving increasing attention globally.[29] Our findings support the need for this approach to increase vaccination completion, including improved supply chains to ensure adequate stocks of vaccines and other supplies, sufficient health workforce, and health facilities that allow for privacy and confidentiality. Capacity building of health workers, including training, retraining, and supportive supervision is also needed.[30] These capacity building approaches should not only focus on technical content, but should support health workers to engage in respectful, empathic, and patient-centered communication.[31] Specifically, better messaging is needed on vaccine availability, scheduling and hours of immunization sessions, common reactions especially for off-schedule vaccines, and policies around issuing new vaccination cards. Involvement of community leaders, religious leaders, and community health workers in those communication strategies may improve vaccination uptake.[32] Ultimately, however, enhanced relationships with patients and the community also require a sufficient and motivated health workforce.[31]

Our findings suggest that re-investment in community-centered vaccine outreach services could improve completion rates. Regularly scheduled outreach events have the potential to greatly facilitate access to immunization services in distant communities, especially when communities are involved in planning them.[33] The WHO and UNICEF have endorsed the RED/REC strategy since 2002, [33,34] but our findings suggest that there has been inadequate resource allocation to implement RED/REC in the study communities.

To successfully implement these recommendations, there is a need for further research on the best channels of communication between health facilities and the communities they serve, as well as on how best to design and implement empathy-building trainings for health workers. Additionally, there is a

For peer review only

need to better understand how to engage husbands and other family members in the vaccination process to reduce the burden on mothers.

Unique strengths of this study include the use of Caregiver Researchers, who drew from their own experiences and familiarity with the community and research topic during data collection and analysis, and the use of multiple data collection methods that generated rich visual, written, and oral data. In addition, centering of the study around the BeSD model ensured that we holistically considered the different categories of vaccination barriers and facilitators.

Our study has several limitations. The specific barriers we identified may not be generalizable to other contexts because influences on vaccine dropout are highly contextual, and determinants relate to social norms, health service policies, and geography. However, the complex nature of compounding barriers may translate to other settings. An additional limitation is that this study was conducted during the COVID-19 pandemic, when many health services were disrupted. While this provided a unique opportunity to examine barriers caused by this crisis, this also may have biased our findings about dropout during non-pandemic times and may have affected the quality of the participatory analysis process, which was conducted virtually. Finally, during recruitment, 13 caregivers were not enrolled due to absence or seasonal relocation to their farms. These caregivers were disproportionately caregivers of PV children, and failure to include them may have influenced the results. Despite these limitations, this study demonstrates that immunization dropout is complex, with caregivers facing different combinations of barriers and facilitators. The findings highlight the need for improved quality and reliability of vaccination services and for community-centered solutions that remove practical barriers and empower caregivers to complete the vaccination process.

Author Contributions

J.P., B.M., A.D., B.C., J.C., and E.L. contributed to study design and logistics. A.D., A.I., B.B., L.J., and Z.M. contributed to data collection. J.P., B.M, D.D., A.I., B.B., L.J., Z.M., M.E., K.B., and E.L. contributed to data analysis. J.P. took the lead in writing the manuscript, with consultation from D.D., K.B., and E.L. All authors provided critical feedback and helped shape the final manuscript.

Acknowledgements

Samira dos Santos assisted with data collection as a Caregiver Researcher. Suzie Truog and Jenny Payne helped review and edit the manuscript.

Competing Interests

None declared.

Funding

For peer review only

This work was supported by Wellcome Trust grant number 219237/Z/19/Z. The funder did not have a role in the study design, analysis, or data interpretation, or manuscript development.

Data sharing statement: No additional data is available.

Figures

Figure 1: Recruitment and enrollment of caregiver participants

For peer review only

REFERENCES

1 UNICEF. Vaccination and Immunization Statistics. 2020.https://data.unicef.org/topic/child-health/immunization/ (accessed 30 Nov 2020).

2 Keja K, Chan C, Hayden G, *et al.* Expanded programme on immunization. *World Health Stat Q Rapp Trimest Stat Sanit Mond* 1988;**41**:59–63.

3 Peck M, Gacic-Dobo M, Diallo MS, *et al.* Global Routine Vaccination Coverage, 2018. *Morb Mortal Wkly Rep* 2019;**68**:937–42. doi:10.15585/mmwr.mm6842a1

4 World Health Organization. Immunization coverage. World Health Organization 2020. https://www.who.int/news-room/fact-sheets/detail/immunization-coverage (accessed 23 Dec 2020).

5 Restrepo-Méndez MC, Barros AJ, Wong KL, *et al.* Inequalities in full immunization coverage: trends in low- and middle-income countries. *Bull World Health Organ* 2016;**94**:794-805B. doi:10.2471/BLT.15.162172

6 World Health Organization. COVID-19 pandemic leads to major backsliding on childhood vaccinations, new WHO, UNICEF data shows. World Health Organ. News. 2021.https://www.who.int/news/item/15-07-2021-covid-19-pandemic-leads-to-major-backsliding-on-childhood-vaccinations-new-who-unicef-data-shows (accessed 17 Aug 2021).

7 Ministry of Health. National Immunization Program Comprehensive Multi-Year Plan (cMYP) 2015-2019. Republic of Mozambique 2014.

8 MISAU. Manual do Programa Alargado de Vacinação. Maputo, Moçambique: : República de Moçambique Ministério da Saúde 2019.

9 Instituto Nacional de Estatistica. INE Destaques. Inst. Nac. Estat. 2021.http://www.ine.gov.mz/ (accessed 28 Jun 2021).

10 Ministério da Saúde- Misau, Instituto Nacional de Estatística- Ine, ICF. Inquérito de Indicadores de Imunização, Malária e HIV/SIDA em Moçambique (IMASIDA) 2015. Published Online First: 1 February 2018.https://dhsprogram.com/publications/publication-AIS12-AIS-Final-Reports.cfm (accessed 24 Feb 2020).

11 Ghosh A, Laxminarayan R. Demand- and supply-side determinants of diphtheria-pertussis-tetanus nonvaccination and dropout in rural India. *Vaccine* 2017;**35**:1087–93. doi:10.1016/j.vaccine.2016.12.024

12 Ateudjieu J, Yakum MN, Goura AP, *et al.* EPI immunization coverage, timeliness and dropout rate among children in a West Cameroon health district: a cross sectional study. *BMC Public Health* 2020;**20**:228. doi:10.1186/s12889-020-8340-6

13 Amimo F, Magit A, Sacarlal J, *et al.* Public health risks of humanitarian crises in Mozambique. *J Glob Health*;**11**:03054. doi:10.7189/jogh.11.03054

For peer review only

14 Favin M, Steinglass R, Fields R, *et al.* Why children are not vaccinated: a review of the grey literature. *Int Health* 2012;**4**:229–38. doi:10.1016/j.inhe.2012.07.004

15 Leung MW, Yen IH, Minkler M. Community based participatory research: a promising approach for increasing epidemiology's relevance in the 21st century. *Int J Epidemiol* 2004;**33**:499–506. doi:10.1093/ije/dyh010

16 World Health Organization. Acceptance and demand. Essent. Program Immun. https://www.who.int/teams/immunization-vaccines-and-biologicals/diseases/tick-borne-encephalitis/essential-programme-on-immunization (accessed 13 May 2021).

17 Government of Zambezia Province. The Province. Portal Gov. Zambezia Prov. 2018.https://www.zambezia.gov.mz/por/A-Provincia (accessed 2 Jun 2021).

18 DPS Zambezia. Relatório anual de 2019 do Programa Alargado de Vacinação. Direcção Provincial de Saúde de Zambézia 2019.

19 Wang C, Burris MA. Photovoice: Concept, Methodology, and Use for Participatory Needs Assessment. *Health Educ Behav* 1997;**24**:369–87. doi:10.1177/109019819702400309

20 Milne E-J, Muir R. Photovoice: A Critical Introduction. In: *The SAGE Handbook of Visual Research Methods*. 1 Oliver's Yard, 55 City Road London EC1Y 1SP: : SAGE Publications, Inc. 2020. 282–96. doi:10.4135/9781526417015.n17

21 Guest G, MacQueen KM, Namey EE. *Applied Thematic Analysis*. SAGE Publications 2011.

22 Rainey JJ, Watkins M, Ryman TK, *et al.* Reasons related to non-vaccination and under-vaccination of children in low and middle income countries: Findings from a systematic review of the published literature, 1999–2009. *Vaccine* 2011;**29**:8215–21. doi:10.1016/j.vaccine.2011.08.096

23 Cobos Muñoz D, Monzón Llamas L, Bosch-Capblanch X. Exposing concerns about vaccination in low- and middle-income countries: a systematic review. *Int J Public Health* 2015;**60**:767–80. doi:10.1007/s00038-015-0715-6

24 Kaufman J, Tuckerman J, Bonner C, *et al.* Parent-level barriers to uptake of childhood vaccination: a global overview of systematic reviews. *BMJ Glob Health* 2021;**6**:e006860. doi:10.1136/bmjgh-2021-006860

25 Merten S, Hilber AM, Biaggi C, *et al.* Gender Determinants of Vaccination Status in Children: Evidence from a Meta-Ethnographic Systematic Review. *PLOS ONE* 2015;**10**:e0135222. doi:10.1371/journal.pone.0135222

26 Jani JV, De Schacht C, Jani IV, *et al.* Risk factors for incomplete vaccination and missed opportunity for immunization in rural Mozambique. *BMC Public Health* 2008;**8**:161. doi:10.1186/1471-2458-8-161

27 Shemwell SA, Peratikos MB, González-Calvo L, *et al.* Determinants of full vaccination status in children aged 12–23 months in Gurùé and Milange districts, Mozambique: results of a population-

For peer review only

based cross-sectional survey. *Int Health* Published Online First: July 2017. doi:10.1093/inthealth/ihx020

28 Tauil M de C, Sato APS, Waldman EA. Factors associated with incomplete or delayed vaccination across countries: A systematic review. *Vaccine* 2016;**34**:2635–43. doi:10.1016/j.vaccine.2016.04.016

29 Hafner T, Shiffman J. The emergence of global attention to health systems strengthening. *Health Policy Plan* 2013;**28**:41–50. doi:10.1093/heapol/czs023

30 Rowe AK, Rowe SY, Peters DH, *et al.* Effectiveness of strategies to improve health-care provider practices in low-income and middle-income countries: a systematic review. *Lancet Glob Health* 2018;**6**:e1163–75. doi:10.1016/S2214-109X(18)30398-X

31 Man JD, Mayega RW, Sarkar N, *et al.* Patient-Centered Care and People-Centered Health Systems in Sub-Saharan Africa: Why So Little of Something So Badly Needed? *Int J Pers Centered Med* 2016;**6**:162–73. doi:10.5750/ijpcm.v6i3.591

32 Goldstein S, MacDonald NE, Guirguis S. Health communication and vaccine hesitancy. *Vaccine* 2015;**33**:4212–4. doi:10.1016/j.vaccine.2015.04.042

33 World Health Organization. Reaching Every District (RED) A guide to increasing coverage and equity in all communities in the African Region. World Health Organization 2017.

34 Mantel C, Cherian T. New immunization strategies: adapting to global challenges. *Bundesgesundheitsblatt Gesundheitsforschung Gesundheitsschutz* 2020;**63**:25–31. doi:10.1007/s00103-019-03066-x

For peer review only

Figure 1: Recruitment and Enrollment of Caregiver Participants

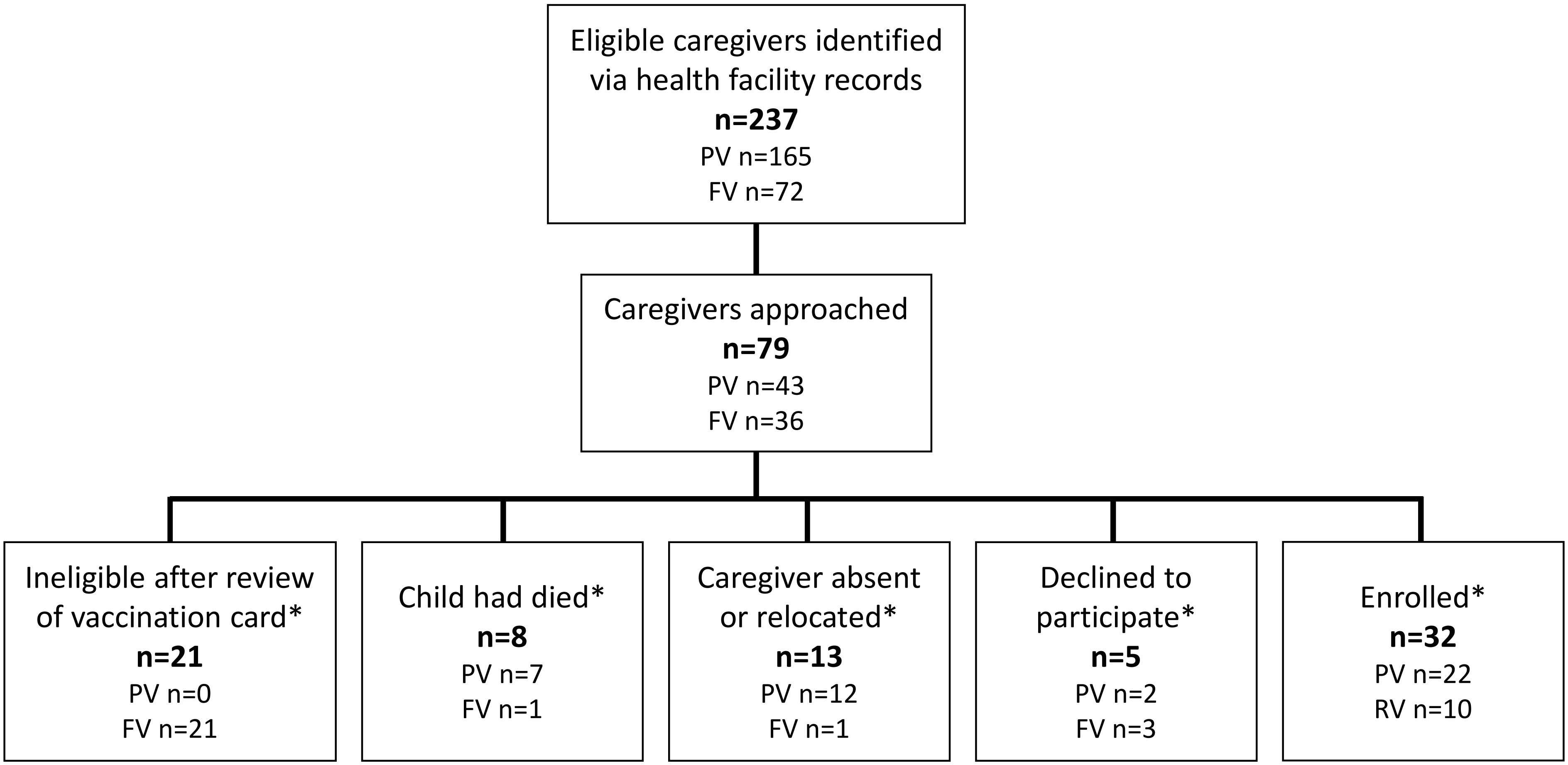

*Vaccination status reflects immunization card, when available

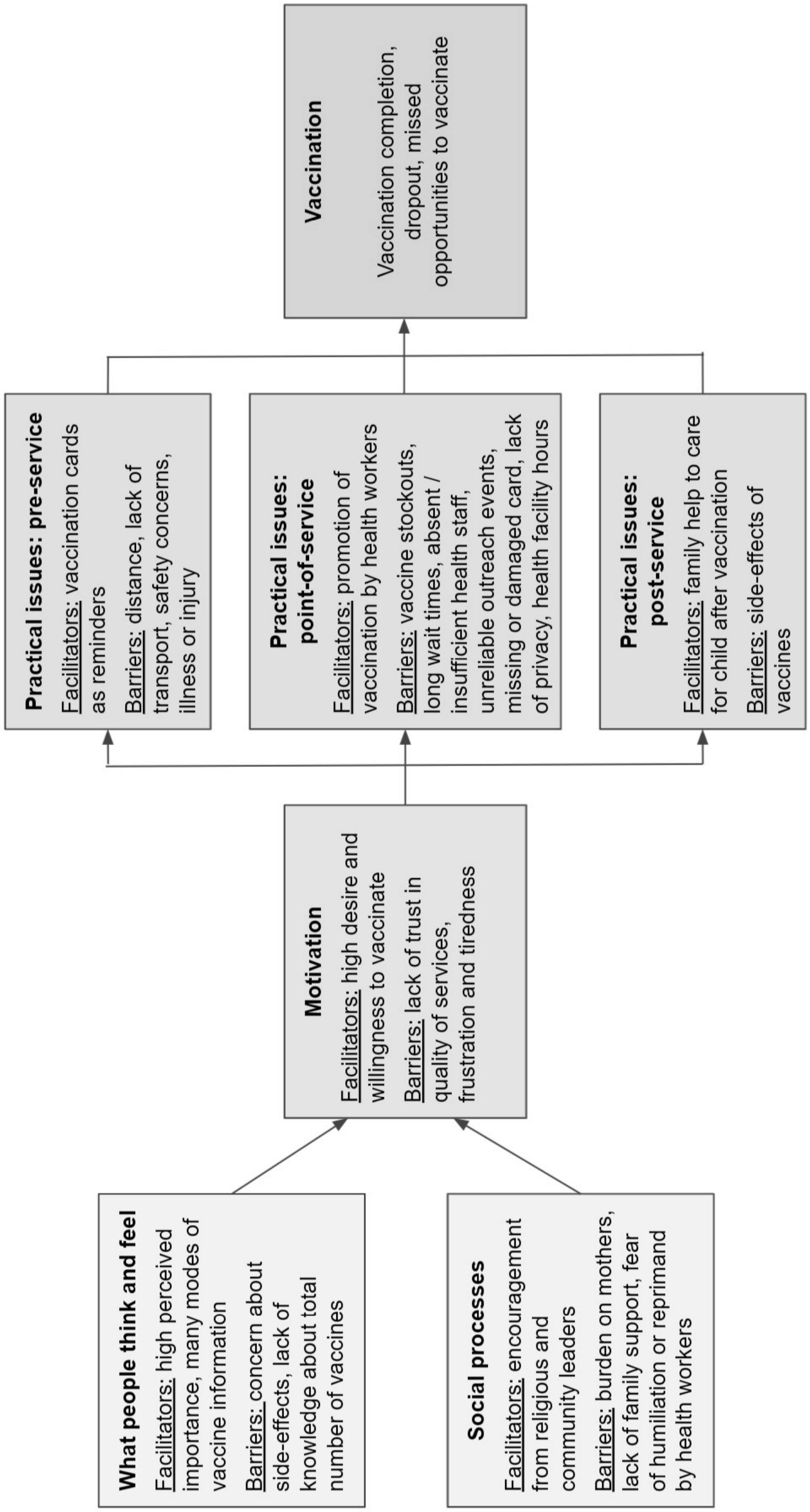

Appendix A: Adapted Increasing Vaccination Model

Appendix B: Mozambique Childhood Immunization Schedule

Vaccine	Dose	Age of administration per Mozambique EPI schedule	
		Ideal	Allowed Range
Tuberculosis	BCG	Birth	Birth – 23 months
Oral Polio	OPV 0	Birth	Birth – 6 weeks
	OPV 1	2 months	6 weeks – 23 months
	OPV 2	3 months	10 weeks – 23 months
	OPV 3	4 months	14 weeks – 23 months
Pentavalent: Diphtheria, Pertussis, Tetanus, Hepatitis B, Haemophilus influenzae type B	DPT-HepB-Hib 1	2 months	6 weeks – 23 months
	DPT-HepB-Hib 2	3 months	10 weeks – 23 months
	DPT-HepB-Hib 3	4 months	14 weeks – 23 months
Pneumococcal conjugate	PCV 1	2 months	6 weeks – 23 months
	PCV 2	4 months	14 weeks – 23 months
	PCV 3	9 months	9 months – 23 months
Rotavirus	RV 1	2 months	6 weeks – 23 months
	RV 2	3 months	10 weeks – 14 weeks
Inactivated Polio	IPV	4 months	14 weeks – 23 months
Measles, Rubella	MR 1	9 months	9 months – 23 months
	MR 2	18 months	18 months – 23 months

For peer review only

Appendix C: Semi-Structured Interview Guide for Caregiver Interviews

Introduction: Good morning/afternoon. I am back to go over the photographs and conduct the remainder of the interview with you. Is now still a good time?

Introduction to in-depth interview:

- This interview will last about 1.5 hours.
- Please know that what you say to us is confidential. We know how important this is to you. We also ask the other participants keep our discussions confidential.
- We would like to record the conversation so that we can document all the information you provide.
- To better understand immunization in your community, it is really important that you share your true beliefs and attitudes towards vaccination, as well as your personal experiences in vaccination of your children.

Informed consent for photos and recording the interview: Now before we get started, I would like to request your informed consent again to review the photographs and record the interview. As mentioned last week, your participation is voluntary and there is no penalty for not taking part. You may refuse to answer any question or withdraw from the study at any time.
Do you have any questions or would you like me to read the informed consent again?

If agree, have them sign the lines on the consent forms for the photographs and recording of the interview!

Discuss "Ground Rules" for the In-depth Interview

- Please don't answer your cell-phone during this interview.
- Do you have any other questions before we get started?

Review of Photovoice:

- Ask them to show the photos they have taken on the camera
- Ask them to pick 3-5 photos that best represent their experience of immunizing [child's name].
- Make sure to pick photographs that are clear!
- **Note: If there are any photos of them, their child or family members, request a signed photo release form by each individual in the photo! If it is the child, they can sign for their child.**
- Upload the selected photos to the computer and paste them in PowerPoint and begin the photovoice session and interview.
    - If the computer isn't working, review the photos on the digital camera and note down the photo number for future reference
- Turn on the recorder!
- Begin the interview using the interview guide

For peer review only

Semi-Structured Discussion Guide for Photovoice In-depth Interview

Instructions: To elicit rich descriptions from participants, when asking follow-up questions or encouraging them to talk more or give you more examples/details, use the following probes:

- *What do you mean by ____ ?*
- *Tell me more about ____.*
- *Can you give me an example of ____ ?*
- *Can you tell me about a time when ____ ?*
- *Can you tell me about the last time ____ ?*
- *Is there anything that made ____ easier?*
- *Is there anything that made ____ harder?*
- *(Can you tell me) Who ____?*
- *(Can you tell me) When ____ ?*
- *(Can you tell me) Where ____ ?*

Probes are completed using only verbatim participant words or phrases.

Interview Guide:

Before we discuss the photos more, can you tell us about how the photovoice process went for you? Did you discuss the task with anyone else in your family or community or have any help in taking the photos?

If yes, Did they have any influence on any of the photos you took?

- If so, in what way?
- Can you provide an example of how they impacted the photos you took and selected?

Once photos are selected, ***ask for each photo:***

- What do we see in this photo?
- What does this represent to you?
- How does this photo represent your vaccination journey?

Now, based on our activity with the photos, we have learned more about your experience, thank you. Now we have some more questions to better understand your experience immunizing your child and the things that influenced [child's name] immunization status. Do you have your child's immunization card which we can refer to during this interview?

1) To start, can you tell me about how people in your community talk about immunizations?
    a) How does this relate to how you feel about immunizing [child's name]?
    b) Has this changed at all with COVID-19?
        i) If yes, why and how?
        ii) If no, why not?

2) Can you describe to me what the experience was like getting immunizations for [child's name]?
    a) How many times did [child's name] get immunizations before the age of two? (look at immunization card)?

For peer review only

b) When did they get these immunizations?
   i) [if they got immunizations more than once] Were these experiences the same? Different?
   ii) How so?
c) Where did [child's name] get their immunizations?
   i) Did they get them all there?
   ii) What did you think about getting the immunizations there?
d) Who immunized [child's name]?
   i) How do you feel about that?
   ii) Did you trust the [health worker/nurse/doctor] who gave the immunizations?
      (1) Why or why not?
e) How satisfied were you with the experience(s) of immunizing [child's name]?
   i) What lead to you feeling this satisfied/dissatisfied?

3) When deciding to immunize [child's name], what things did you consider?
   a) Was there anyone who helped you make that decision?
      i) Who? How did they help?
   b) Did you intend for your child to be fully vaccinated according to the recommended schedule?
      i) If yes, why?
      ii) If no, why not?
   c) Did you have questions about the recommendations for immunizing [child's name]?
      i) Why or why not?
   d) How important did you feel getting these immunizations was, if at all, for [child's name]?
   e) Have things changed since COVID-19?
      i) If so, how?

4) What kind of information, if at all, did you receive about immunizing your child or about immunizations in general?
   a) When did you receive this information?
   b) Where did this information come from?
      i) Health center?
      ii) Social media (Facebook, WhatsApp)?
      iii) Neighbours?
   c) How often, if at all, did you receive information?
   d) To what extent did you trust this information?
   e) Did you get information about how many immunizations your child needed and when they were needed?
   f) Did this change with COVID-19? How?

For peer review only

5) [for those who have a child who dropped out] Can you describe why [child's name] didn't receive all their vaccinations by age two?
    a) What factors do you think contributed to that?
        i) Probe on:
            (1) What kind of information did you receive about your child's immunization schedule, if at all?
            (2) Did you know what your child's immunization schedule was?
            (3) COVID-19
    b) What was hard about trying to immunize [child's name]?
        i) Can you provide an example?
        ii) How, if at all, has this changed since COVID-19?
    c) What was easy about trying to immunize [child's name]?
        i) Can you provide an example?
        ii) How, if at all, has this changed since COVID-19?

6) [for those who have a fully-immunized child] What factors do you think contributed to being able to fully immunize [child's name] by the age of two?
    a) What was hard about trying to immunize [child's name]?
        i) Can you provide an example?
        ii) How, if at all, has this changed since COVID-19?
    b) What was easy about trying to immunize [child's name]?
        i) Can you provide an example?
        ii) How, if at all, has this changed since COVID-19?

7) How do you wish the experience of getting all the recommended immunizations for [child's name] could be easier, if at all?
    a) What would you change?
    b) What would you keep the same?
    c) How would these changes look in times where there is no COVID-19?
    d) How does it look now with COVID-19?

Conclusion: Are there any final thoughts you wish to share about your experiences with immunizing your 2-3 year old child?

We would like to thank you for taking the time to discuss this important topic with us!

For peer review only

Appendix D: Semi-Structured Interview Guide for Health Worker Interviews

Introduction to Structured Phone Interview: The purpose of the debrief activities is to reflect on and discuss your messages with the healthcare workers you corresponded with. You will use the message notes to debrief with the healthcare workers about the immunization process. This is a rough guide of questions to ask the healthcare workers, and additional questions or topics can be discussed based on specific observations that you made from the messages.

Interview Questions

1) Now, I am going to walk through some of the activities you mentioned in your messages, and I would like to get your perspective on:
   a) Were these typical [experiences, processes, observations]?
      i) If so, how? If not, how did they vary from normal?
   b) Is there anything else you would like to share or explain about the [experience, process, observation] that may not have been captured in the messages?

2) How do you see your role in immunizations?
   a) How do you feel about that role?
   b) Do you feel supported in your role? If not, why? If yes, how so?
   c) How do you generally feel about the tasks associated with immunizations?

3) How do you feel about how immunization records are reported?
   a) How do you feel about overall system for immunizations?
   b) What about management of immunizations?

4) Now, I would like to get a better sense of your work around immunizing children under the age of two. Can you walk through a typical day of conducting immunizations for children under the age of two? [Probe on what activities they do to prepare for immunizations, conduct immunizations, document immunizations, educate around immunization]
   For each step/activity?
   a) Why do you take that particular step?
   b) Who is typically involved in that step?
   c) What you feel when going through that step?
   d) What factors make that step easier/harder?
   e) Can you guide me through the steps that you took today when administering a vaccination?
   f) Are there any cases where you would not administer a vaccination to a child? Why or why not?

5) Are there other, non-typical ways that you provide immunizations?
   a) Can you tell me about those?

For peer review only

6) Outside of conducting immunizations, what else do you do?
    a) Can you tell me about how under-two vaccinations fit into this workload?

7) What is challenging about administering under-two vaccinations?
    a) Did you encounter any of those challenges over the past month? If so, which ones? How did that make your job more challenging?

8) What factors make it easier for you to administer vaccinations?
    a) Did you encounter any of those factors over the past month? If so, which ones? How did that make your job easier?

9) What are your personal beliefs around vaccinations?

10) Can you describe the conversations you usually have with caregivers while administering vaccines?
    a) What about how you see your role in providing advice or convincing caregivers to vaccinate their children under the age of two?

11) What do you view as the primary drivers of under-two immunization drop-outs?
    a) Can you provide an example?

For peer review only

Appendix E: Photovoice Instructional Materials

A CÂMERA

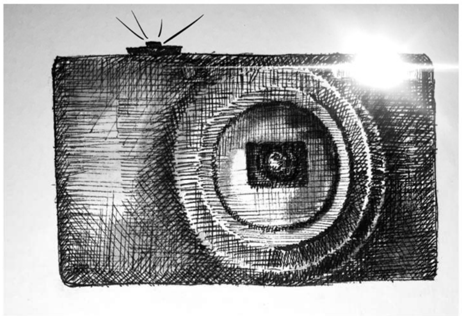

1. Frente da câmera

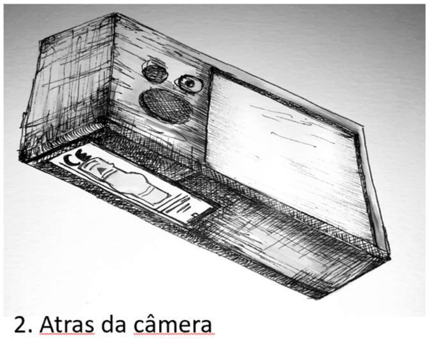

2. Atras da câmera

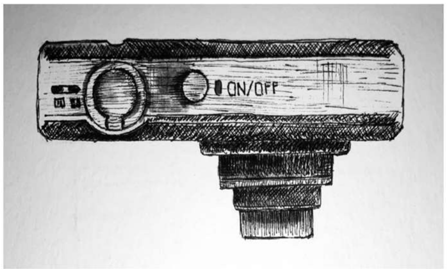

3. Parte superior da câmera

SEGURAR A CÂMERA PARA TIRAR PHOTOS

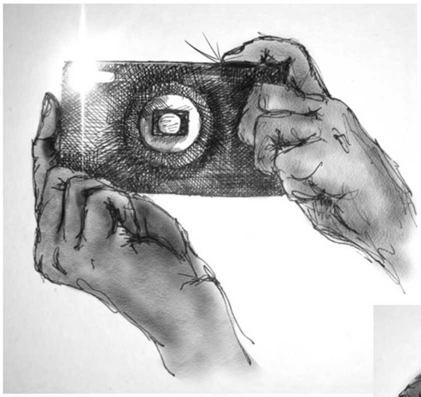

1. Uma Maneira

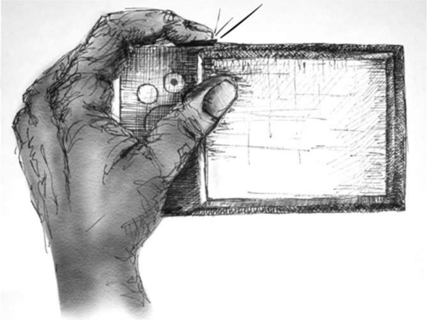

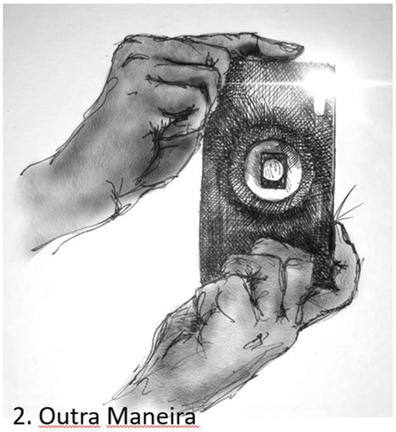

2. Outra Maneira

BATERIA DA CÂMERA

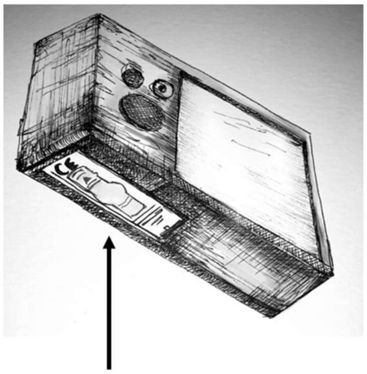

1. Localização da bateria

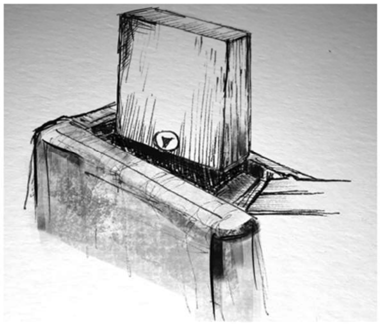

2. Abra o local da bateria

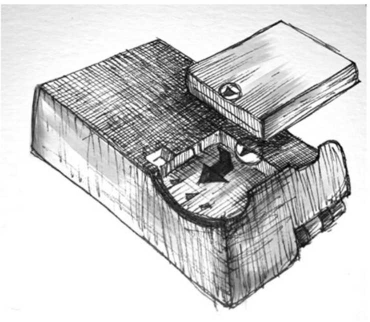

3. Carrega a bateria no carregador

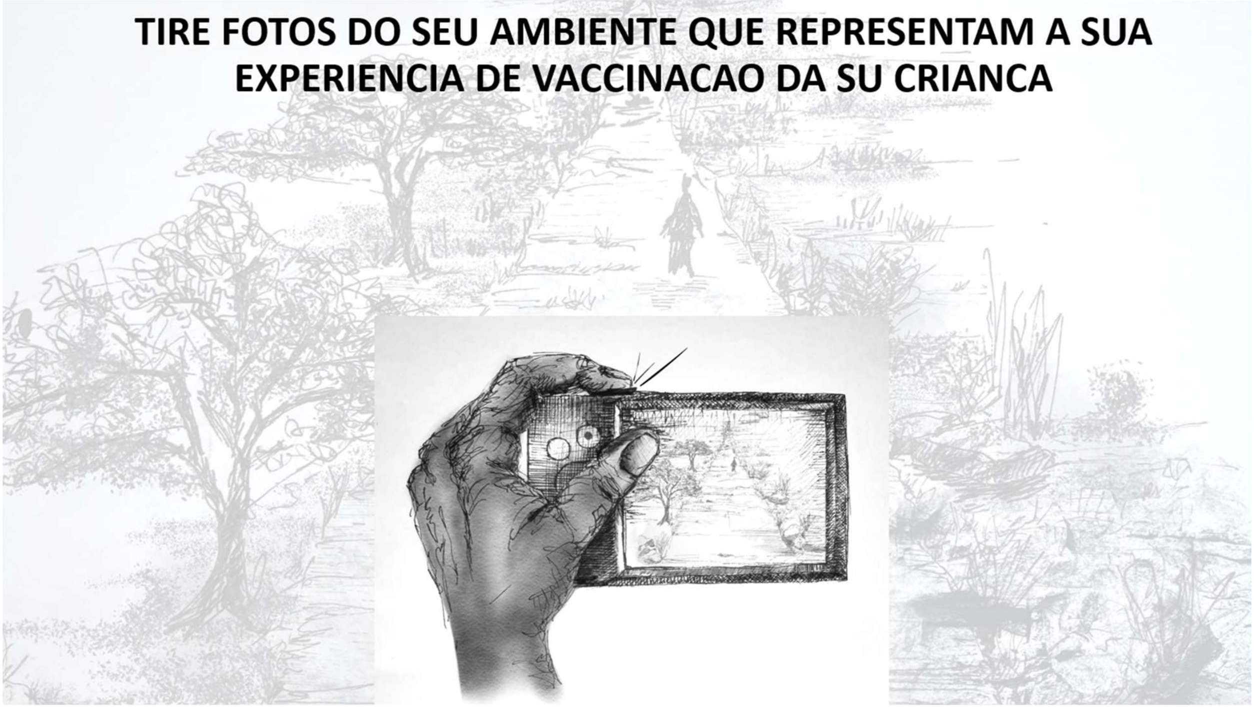

Standards for Reporting Qualitative Research (SRQR)*

http://www.equator-network.org/reporting-guidelines/srqr/

Title and abstract

	Page/line no(s).
**Title** - Concise description of the nature and topic of the study Identifying the study as qualitative or indicating the approach (e.g., ethnography, grounded theory) or data collection methods (e.g., interview, focus group) is recommended	2/3-4
**Abstract** - Summary of key elements of the study using the abstract format of the intended publication; typically includes background, purpose, methods, results, and conclusions	3/3-38

Introduction

**Problem formulation** - Description and significance of the problem/phenomenon studied; review of relevant theory and empirical work; problem statement	4/32-52
**Purpose or research question** - Purpose of the study and specific objectives or questions	4/50-52

Methods

**Qualitative approach and research paradigm** - Qualitative approach (e.g., ethnography, grounded theory, case study, phenomenology, narrative research) and guiding theory if appropriate; identifying the research paradigm (e.g., postpositivist, constructivist/ interpretivist) is also recommended; rationale**	5/5-18
**Researcher characteristics and reflexivity** - Researchers' characteristics that may influence the research, including personal attributes, qualifications/experience, relationship with participants, assumptions, and/or presuppositions; potential or actual interaction between researchers' characteristics and the research questions, approach, methods, results, and/or transferability	5/9-13<br>6/8-15<br>7/41-45
**Context** - Setting/site and salient contextual factors; rationale**	5/22-29
**Sampling strategy** - How and why research participants, documents, or events were selected; criteria for deciding when no further sampling was necessary (e.g., sampling saturation); rationale**	5/33-52
**Ethical issues pertaining to human subjects** - Documentation of approval by an appropriate ethics review board and participant consent, or explanation for lack thereof; other confidentiality and data security issues	6/22-25<br>7/47-51
**Data collection methods** - Types of data collected; details of data collection procedures including (as appropriate) start and stop dates of data collection and analysis, iterative process, triangulation of sources/methods, and modification of procedures in response to evolving study findings; rationale**	6/17 – 7/7

For peer review only - http://bmjopen.bmj.com/site/about/guidelines.xhtml

**Data collection instruments and technologies** - Description of instruments (e.g., interview guides, questionnaires) and devices (e.g., audio recorders) used for data collection; if/how the instrument(s) changed over the course of the study	6/17-21
**Units of study** - Number and relevant characteristics of participants, documents, or events included in the study; level of participation (could be reported in results)	8/3-8<br>9/8-11
**Data processing** - Methods for processing data prior to and during analysis, including transcription, data entry, data management and security, verification of data integrity, data coding, and anonymization/de-identification of excerpts	6/44-46<br>7/6-7<br>7/11-22
**Data analysis** - Process by which inferences, themes, etc., were identified and developed, including the researchers involved in data analysis; usually references a specific paradigm or approach; rationale**	7/23-38
**Techniques to enhance trustworthiness** - Techniques to enhance trustworthiness and credibility of data analysis (e.g., member checking, audit trail, triangulation); rationale**	7/14-16<br>7/28-29

Results/findings

**Synthesis and interpretation** - Main findings (e.g., interpretations, inferences, and themes); might include development of a theory or model, or integration with prior research or theory	9/42-10/24<br>Pages 10-15
**Links to empirical data** - Evidence (e.g., quotes, field notes, text excerpts, photographs) to substantiate analytic findings	Pages 10-15

Discussion

**Integration with prior work, implications, transferability, and contribution(s) to the field -** Short summary of main findings; explanation of how findings and conclusions connect to, support, elaborate on, or challenge conclusions of earlier scholarship; discussion of scope of application/generalizability; identification of unique contribution(s) to scholarship in a discipline or field	15/35-16/51
**Limitations** - Trustworthiness and limitations of findings	17/13-29

Other

**Conflicts of interest** - Potential sources of influence or perceived influence on study conduct and conclusions; how these were managed	17/49-51
**Funding** - Sources of funding and other support; role of funders in data collection, interpretation, and reporting	18/3-4

*The authors created the SRQR by searching the literature to identify guidelines, reporting standards, and critical appraisal criteria for qualitative research; reviewing the reference lists of retrieved sources; and contacting experts to gain feedback. The SRQR aims to improve the transparency of all aspects of qualitative research by providing clear standards for reporting qualitative research.

For peer review only

For peer review only - http://bmjopen.bmj.com/site/about/guidelines.xhtml

**The rationale should briefly discuss the justification for choosing that theory, approach, method, or technique rather than other options available, the assumptions and limitations implicit in those choices, and how those choices influence study conclusions and transferability. As appropriate, the rationale for several items might be discussed together.

Reference:

O'Brien BC, Harris IB, Beckman TJ, Reed DA, Cook DA. **Standards for reporting qualitative research: a synthesis of recommendations.** *Academic Medicine*, Vol. 89, No. 9 / Sept 2014 DOI: 10.1097/ACM.0000000000000388

For peer review only

For peer review only - http://bmjopen.bmj.com/site/about/guidelines.xhtml
